# Identification of Differential Proteins in Thrombi of Cardioembolic and Atherothrombotic Etiology in Patients with Ischemic Stroke

**DOI:** 10.3390/ijms26178333

**Published:** 2025-08-28

**Authors:** Lorena Peracho, Emma Martínez-Alonso, Isabel Bermúdez, Antonio Cruz-Culebras, Alicia De Felipe, Eduardo Fandiño, Sebastián García-Madrona, María Consuelo Matute-Lozano, Jose Carlos Méndez-Cendón, Rocío Vera-Lechuga, Jaime Masjuan, Alberto Alcázar

**Affiliations:** 1 Proteomics Unit, IRYCIS, Hospital Universitario Ramón y Cajal, 28034 Madrid, Spain; 2 Department of Research, IRYCIS, Hospital Universitario Ramón y Cajal, 28034 Madrid, Spain; 3 Interventional Neuroradiology Unit, Department of Radiology, Hospital Universitario Ramón y Cajal, 28034 Madrid, Spain; 4 Department of Neurology, IRYCIS, Hospital Universitario Ramón y Cajal, 28034 Madrid, Spain; 5 Department of Neurology, Facultad de Medicina, Universidad de Alcalá, 28801 Alcalá de Henares, Spain

**Keywords:** biomarker, diagnostic, ischemic stroke, proteomics, thrombus

## Abstract

Knowing the precise etiology in ischemic stroke is necessary to ensure accurate diagnosis and decide on appropriate preventive treatments, especially in those of undetermined cause. Analysis of the thrombus protein composition could be useful to identify diagnostic biomarkers to help determine the stroke origin. Thrombi from 54 ischemic stroke patients with large vessel occlusion (LVO), of cardioembolic and atherothrombotic etiology, were analyzed using a proteomics approach. The proteome profile was compared between them to detect differential proteins of each etiology. Peptides of those differential proteins were quantified and related to the neurological function and clinical status of the patients. Of the 516 proteins identified, three showed significant differences between atherothrombotic and cardioembolic thrombi. These were fibronectin (FINC), 2,3-bisphosphoglycerate mutase (PMGE), and tropomyosin-1 (TPM1). Combining these proteins in a biomarker panel provided good sensitivity and high specificity for differentiating cardioembolic and atherothrombotic strokes. In addition, several of the quantified peptide levels correlated with clinical parameters related to stroke severity and prognosis. Three proteins differentially detected in ischemic stroke thrombi could be useful tools for accurately diagnosing ischemic stroke etiology, particularly in cases of undetermined cause. These biomarkers should be further analyzed in prospective multicenter studies to demonstrate their usefulness.

## 1. Introduction

Stroke is a global public health issue that remains one of the leading causes of death and disability in the world [1,2], although stroke death rates have declined significantly in the last years in developed countries [3]. Clinically, stroke is characterized by a sudden loss of neurological function resulting from a rapid decline in cerebral blood flow (CBF) [4]. The risk factors associated with stroke are diverse and include age, sex, and race/ethnicity, as well as modifiable factors such as hypertension, smoking, obesity, alcohol consumption, diabetes mellitus, low physical activity, atrial fibrillation (AF), and sleep apnea [5]. Notably, modifiable risk factors are of great importance as they account for 90% of the overall stroke risk [6].

Large vessel occlusions (LVOs), which involve arteries such as the vertebral, basilar, carotid, and middle and anterior cerebral arteries, account for approximately 38% of ischemic strokes and are associated with a worse prognosis when compared to non-LVO ischemic strokes [7]. The disruption of CBF triggers neuronal damage through a cascade of complex pathophysiological processes, including excitotoxicity, oxidative stress, neuroinflammation, protein synthesis inhibition, and various mechanisms of cell death [8]. The extent of damage is influenced by the duration, severity, and location of the ischemia. The affected brain tissue can be categorized into two regions: the core, where tissue damage is irreversible, and the surrounding area, known as the ischemic penumbra [9].

Ischemic strokes can arise from either thrombotic or embolic events. Thrombotic strokes occur when a vessel is obstructed by a thrombus formed at the site of the occlusion, often due to vascular conditions such as atherosclerosis or dissection. Embolic strokes are caused by a thrombus that migrates from its original location to a cerebral artery, obstructing CBF and leading to ischemia. Embolic sources can originate from the heart, aorta or have an unknown origin [10].

Given the heterogeneous etiology of ischemic stroke, accurate etiological classification is crucial for ensuring precise diagnosis, guiding the selection of the most appropriate preventive treatments, and reducing the risk of recurrence. One of the most widely used classifications is the TOAST classification (Trial of Org 10172 in Acute Stroke Treatment), which categorizes ischemic stroke into five subtypes: (i) large artery atherosclerosis (LAA), (ii) cardioembolism (CE), (iii) small vessel occlusion, (iv) stroke of undetermined etiology, and (v) stroke of infrequent etiology [11,12]. Each subtype corresponds to distinct pathophysiological mechanisms and requires specific secondary prevention strategies, issues that can be particularly difficult in cases of undetermined cause.

The primary therapeutic goal in patients with acute ischemic stroke is the prompt restoration of CBF by removing the occlusion through recanalization and reperfusion [13]. This approach aims to minimize tissue damage in brain areas that are still salvageable. Currently, two main therapeutic options have been approved for the treatment of ischemic stroke: intravenous thrombolysis with tissue plasminogen activator (tPA and tenecteplase) and endovascular interventions, such as mechanical thrombectomy [14]. Due to the limitations of fibrinolytic therapy alone and the low recanalization rates in LVOs [15], mechanical thrombectomy (MT) has emerged as an effective treatment option for acute ischemic stroke. MT has been used since the early 21st century to treat various conditions, but it was not until 2015 that several clinical trials demonstrated its safety and efficacy, consolidating it as a treatment for ischemic stroke, either in combination with or independent of thrombolytic therapy [16,17,18,19,20].

MT usually involves the use of a stent retriever, a device inserted through a catheter to mechanically extract the thrombus from the occluded artery. The procedure is commonly performed via access through the femoral artery, advancing the catheter to the site of the occlusion. The stent retriever is then deployed to capture and remove the clot [21].

One of the advantages of MT is that it allows for the extraction of the thrombus, providing an opportunity for researchers to study its composition. Understanding the composition of thrombi holds the potential to enhance strategies for thrombolysis and mechanical thrombectomy, addressing the existing limitations of these therapies. Recent research has shown that thrombi with a higher proportion of red blood cells are associated with more efficient recanalization and better clinical outcomes compared to thrombi predominantly composed of fibrin [22,23,24]. Moreover, fibrin-rich thrombi tend to be more resistant to both pharmacological and mechanical recanalization [25].

Comprehending the molecular composition of thrombi is important for understanding the etiology of the thrombus, which can help tailor secondary prevention strategies. This can offer valuable insights into the underlying pathogenesis of ischemic stroke, thereby guiding the selection of anticoagulants or antiplatelet agents for secondary prevention. It is here that the knowledge of the etiology of ischemic stroke of undetermined cause acquires all its importance for its subsequent preventive treatment. Thus, the histological composition of thrombi has been extensively studied, although findings have not always been consistent [24,26,27,28]. The utilization of emerging “omics” technologies, including proteomics, presents promising avenues for studying the molecular constituents of thrombi, identifying biomarkers, and distinguishing between different etiologies of ischemic stroke.

This study analyzes the protein composition of thrombi in ischemic stroke to determine whether it can provide information about the cause of the stroke. We hypothesize that the characterization of the protein composition of the thrombus could provide information on the origin of the stroke and aid in the identification of biomarkers that could be useful for the diagnosis of ischemic strokes of undetermined cause and for therapeutic decision-making. In this study, we performed a differential proteomic analysis comparing thrombi of cardioembolic and atherothrombotic origin. The results of this study identified molecular differences leading to the finding of three biomarkers, which could be useful in determining the etiology of stroke.

## 2. Results

### 2.1. Patient Characteristics

In this study were included 54 ischemic stroke patients, who were categorized into two groups based on ischemic stroke subtype, cardioembolic and atherothrombotic, with 27 patients in each group. Of these patients, 31 (57.4%) were male, and 23 (42.6%) were female. The overall median age was 71 years, with a significantly higher median age of 78 years in the cardioembolic group compared to 64 years in the atherothrombotic group (*p* = 0.002). This age difference may be attributed to the increased incidence of atrial fibrillation (AF) in older populations, which predisposes them to cardioembolic strokes. As expected, AF was significantly more prevalent in the cardioembolic group, consistent with its role as a major risk factor for cardioembolic stroke.

Common risk factors for ischemic stroke in the study population included hypertension (66.7%), hyperlipidemia (79.6%), and smoking (25.9%). Notably, smoking was significantly more prevalent in the atherothrombotic group compared to the cardioembolic group (*p* = 0.004), which is consistent with smoking’s established role in promoting atherosclerosis and thrombus formation.

Regarding treatment, 11 patients in the cardioembolic group and 17 patients in the atherothrombotic group received intravenous fibrinolysis with recombinant tPA (rtPA), with no significant difference between the two groups (*p* = 0.173). Those patients who underwent precision stenting were fully atherothrombotic.

The median NIHSS score on admission was 18 (range: 9–26) in the cardioembolic group and 15 (range: 3–23) in the atherothrombotic group. By discharge, both groups showed significant clinical improvement, with the median NIHSS score dropping to 4 in both the cardioembolic and atherothrombotic groups.

The demographic, clinical, and treatment details of the participating patients are summarized in Table 1.

### 2.2. Biomarker Identification

**Figure 1:**
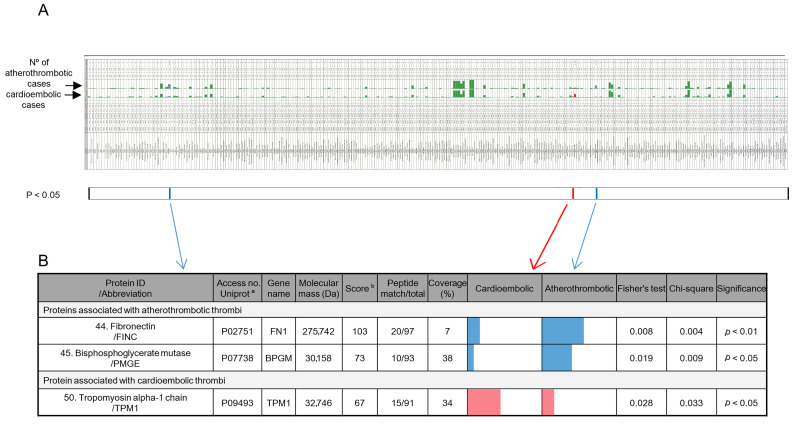
Identification of differential proteins in thrombi of cardioembolic and atherothrombotic etiology. (**A**) Graphical representation of the table of all protein identifications. Green bars indicate the number of atherothrombotic or cardioembolic thrombi in which the protein was identified. The complete table is available in Supplementary Table S1. (**B**) Proteins with significant differences between cardioembolic and atherothrombotic etiology found in the complete identified proteome. Colored bars represent the number of cardioembolic or atherothrombotic thrombi in which the protein was identified: blue bars indicate proteins significantly identified in atherothrombotic thrombi, and red bars indicate proteins significantly identified in cardioembolic thrombi. Statistical analyses were performed using the chi-squared and Fisher’s exact tests, with significance set at *p* < 0.05. ^a^ Accession numbers from the UniProt database (https://www.uniprot.org/, accessed on 30 April 2025). ^b^ Protein scores > 56 were considered significant (*p* < 0.05) based on peptide mass fingerprinting using the Mascot database search algorithm (Matrix Science, London, UK, https://www.matrixscience.com/, accessed on 30 April 2025).

This study aims to identify potential biomarkers that could distinguish between cardioembolic and atherothrombotic thrombi. These biomarkers may serve as valuable tools for therapeutic decision-making and the prevention of secondary strokes. To this end, a proteomic study of the 54 thrombi was performed. From this analysis, a total of 2570 protein identifications were obtained, corresponding to 516 different proteins (Figure 1A and Supplementary Table S1). Table S1 shows the protein identification data obtained from the mass spectrometry analysis. To assess the differences in protein identification, a contingency table was constructed with two categorical variables: the presence or absence of a protein and the type of thrombi (cardioembolic or atherothrombotic). The results of the statistical analysis, using Fisher’s exact test and chi-squared test for all identified proteins, are also shown in Table S1. Most of the proteins did not show significant differences, suggesting no association between the presence of these proteins and the classification of stroke subtype. Interestingly, three proteins showed statistical differences between cardioembolic and atherothrombotic thrombi (*p* < 0.05) (Figure 1B and Table S1). These proteins, identified as biomarkers, were tropomyosin alpha-1 chain (TPM1), in the cardioembolic group; and fibronectin (FINC) and 2,3-bisphosphoglycerate mutase (PMGE), in the atherothrombotic group.

### 2.3. Quantification of Biomarkers

To assess and validate these biomarkers, we analyzed the relative abundance of these proteins through the quantification of their peptides. TPM1, FINC, and PMGE peptide quantification could aid in differentiating stroke etiology, potentially contributing to more personalized treatment approaches. In this study, the most representative peptides of each of the identified biomarkers were considered for quantification, being a representative peptide the one that was identified in at least three different thrombi.

**Figure 2:**
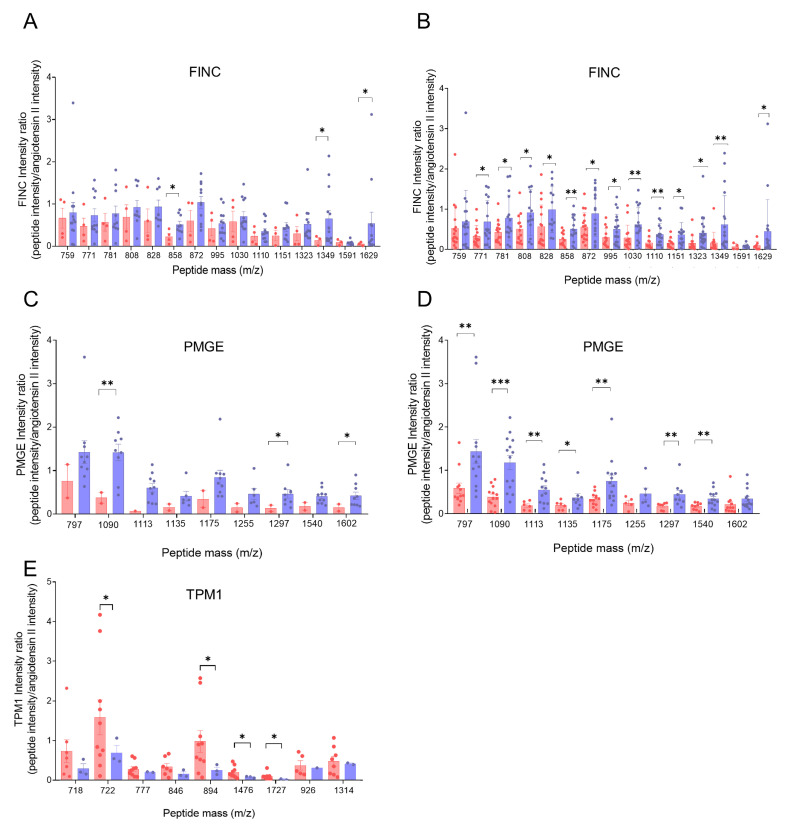
Peptide quantification of biomarkers from cardioembolic (red) and atherothrombotic (blue) thrombi analyzed by mass spectrometry. Peptides were quantified relative to the angiotensin-II peptide (internal standard) using the peptide/angiotensin-II intensity ratio. (**A**) Quantification of FINC peptides, with MH+ mass peaks (peptides) at *m*/*z* 759, 771, 781, 808, 828, 858, 872, 995, 1030, 1110, 1151, 1323, 1349, 1591, and 1629, in thrombi with identified FINC. (**B**) Quantification of FINC peptides in all thrombi, including those in which FINC was not identified. (**C**) Quantification of PMGE peptides, with MH+ mass peaks (peptides) at *m*/*z* 797, 1090, 1113, 1135, 1175, 1255, 1297, 1540, and 1602, in thrombi with identified PMGE. (**D**) Quantification of PMGE peptides in all thrombi, including those in which PMGE was not identified. (**E**) Quantification of TPM1 peptides, with MH+ mass peaks (peptides) at *m*/*z* 718, 722, 846, 894, 1476, 1727, 926, and 1314. Graphs show peptide levels for cardioembolic and atherothrombotic thrombi. Data are shown as individual values (dots) and means represented as bars with error bars indicating SE. Significance was set at * *p* < 0.05, ** *p* < 0.01, and *** *p* < 0.001, and the cardioembolic group was compared to the atherothrombotic group by Student’s *t*-test.

FINC peptide quantification revealed higher levels in the atherothrombotic thrombus group compared with cardioembolic thrombi, with mass peptides (*m*/*z*) 858, 1349, and 1629 showing significant differences (Figure 2A). An extended analysis that included the detection of each peptide mass in all of the thrombi of each respective group confirmed this trend, with multiple peptides showing significant differences between groups (Figure 2B). Notably, mass peptides 858, 1030, 1110, and 1349 had *p*-values < 0.01. Similarly, PMGE peptides were found to have higher levels in the atherothrombotic thrombus group, with mass peptides 1090, 1297, and 1602 showing significant differences (Figure 2C). In the extended analysis, including the peptide mass found in all of the thrombi of each respective group, additional peptides showed significant differences, including mass peptides 797, 1113, 1175, 1297, 1540 (*p* < 0.01), and 1090 (*p* < 0.001) (Figure 2D). For TPM1, peptide levels were higher in the cardioembolic thrombi compared to the atherothrombotic thrombi. To ensure accurate quantification, only samples in which TPM1 was unequivocally identified were included, minimizing the risk of misquantifying other tropomyosins. Mass peptides 722, 894, 1476, and 1727 showed significantly higher levels in the cardioembolic thrombus group compared with the atherothrombotic thrombi (Figure 2E).

In summary, the quantification of the peptides of the proteins TPM1, FINC, and PMGE supports the results of the identified biomarkers (see above), showing increased presence and higher levels of TPM1 in cardioembolic thrombi, and of FINC and PMGE in atherothrombotic thrombi.

Finally, to evaluate the ability of each of the peptides to discriminate between cardioembolic and atherothrombotic patients, ROC (receiver operator characteristic) curve analysis was performed. The AUC values ranged from 0.5 to 0.7, indicating the limited discriminatory capacity of the peptides.

### 2.4. Correlation of Biomarkers with Clinical Outcomes in Thrombus Analysis

**Figure 3:**
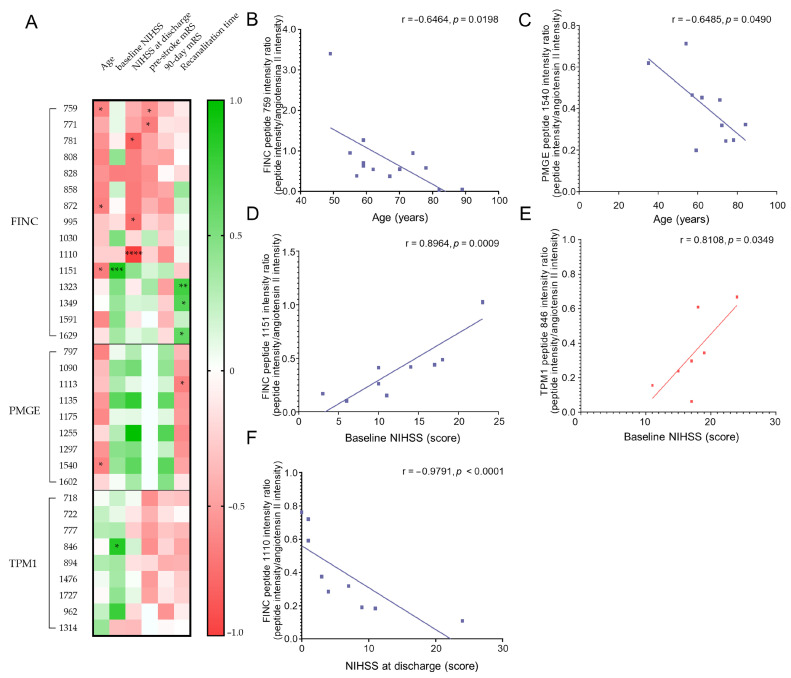
Correlation of biomarkers with stroke severity and prognosis. (**A**) Heat map showing the correlation between the quantified levels of each biomarker peptide and clinical parameters related to stroke severity and prognosis. Red and green indicate positive and negative correlations, respectively, and color intensity represents the Spearman rank correlation coefficient (rho) (* *p* < 0.05, ** *p* < 0.01, *** *p* < 0.001, and **** *p* < 0.0001). (**B**–**F**) Scatter plots with fitted lines for significant correlations between quantified peptide levels and the following variables: (**B**) FINC peptide of mass at *m*/*z* 759 and age (Spearman’s rho = −0.6464, *p* = 0.0198); (**C**) PMGE peptide of mass at *m*/*z* 1540 and age (Spearman’s rho = −0.6485, *p* = 0.0490); (**D**) FINC peptide of mass at *m*/*z* 1151 and baseline NIHSS score (Spearman’s rho = 0.8964, *p* = 0.0009); (**E**) TPM1 peptide of mass at *m*/*z* 846 and baseline NIHSS score (Spearman’s rho = 0.8108, *p* = 0.0349); and (**F**) FINC peptide of mass at *m*/*z* 1110 and NIHSS score at discharge (Spearman’s rho = −0.9791, *p* < 0.0001).

We evaluated the potential prognostic value of candidate biomarkers found in thrombi and explored their potential clinical relevance. To achieve this, we analyzed the correlation between the quantification of the peptides and the clinical variables or patient outcomes in each cardioembolic and atherothrombotic group. Correlations were performed between the quantified levels of each biomarker peptide and the clinical variables of patients whose thrombi were positive for that specific biomarker (Figure 3A).

Correlation with age. A negative correlation was observed between FINC levels and patient age in atherothrombotic thrombi. Specifically, peptides 759, 872, and 1151 exhibited significant correlations, with Spearman correlation coefficients (r) around −0.65 (Figure 3B). These negative correlations indicate that FINC levels in atherothrombotic thrombi tend to decrease with advancing patient age. Similarly, PMGE levels in atherothrombotic thrombi showed a significant negative correlation with age, notably with peptide 1540 (Figure 3C). This finding indicates that, similarly to FINC, PMGE levels decrease with advancing patient age, suggesting that both proteins may be affected by aging processes in atherothrombotic stroke patients.

Correlation with baseline NIHSS score. A positive correlation was observed between FINC levels and baseline NIHSS scores in atherothrombotic thrombi, with peptide 1151 showing a strong and significant correlation (r ~ 0.9, Figure 3D). These findings suggest that higher FINC levels are associated with more severe clinical status at the onset of ischemic stroke, indicating that FINC may serve as a potential marker for initial brain damage. Furthermore, a significant correlation was observed between TPM1 peptide 846 and baseline NIHSS scores (r ~ 0.8, Figure 3E), indicating that higher levels of TPM1 are associated with worse clinical status in cardioembolic patients at the onset of stroke. This suggests that TPM1 may serve as a potential marker of initial brain damage in this context.

Correlation with NIHSS score at discharge. A negative correlation was observed between FINC peptide levels and NIHSS scores at discharge in atherothrombotic patients. Specifically, peptides 781, 995, and 1110 exhibited significant negative correlations with high coefficients (r ~ −0.9, Figure 3F). These results suggest that higher FINC levels are associated with improved clinical outcomes at discharge, potentially indicating a reparative role for FINC in stroke recovery. However, elevated levels at stroke onset could also reflect initial brain damage, as indicated by correlations with baseline NIHSS scores (see above). This would suggest a possible dual role for FINC: as a marker of damage at the onset of the disease, and as a marker of recovery after the acute phase.

Correlation with other clinical variables. When correlating FINC levels with pre-stroke mRS scores in atherothrombotic thrombi, peptides 759 and 771 showed significant negative correlations (r ~ −0.6, Figure 3A). Additionally, negative correlations were found between FINC levels and mRS scores at three months post-discharge, notably peptides 858, 872, 995, and 1110 (r ~ −0.6, Figure 3A). These results suggest that higher FINC levels are associated with better patient outcomes both before and after the ischemic event.

A positive correlation was observed between FINC levels and recanalization time, with peptides 1323, 1349, and 1629 showing significant correlations (r ~ 0.7, Figure 3A). The positive correlation with recanalization time implies that elevated FINC levels may reflect thrombus stability, influencing its progression.

For PMGE, a negative correlation was observed between peptide levels and recanalization time in atherothrombotic patients, with peptide 1113 showing a significant correlation (r ~ −0.7, *p* = 0.0438). These findings suggest that longer recanalization times are associated with lower PMGE levels in atherothrombotic thrombi, highlighting the impact of thrombus extraction time on PMGE levels.

In summary, these findings suggest that the biomarkers FINC, PMGE, and TPM1 hold prognostic potential in stroke patients, particularly for differentiating between cardioembolic and atherothrombotic origins and predicting patient outcomes. However, further research is required to validate these correlations and assess their clinical utility in patient management and treatment strategies.

### 2.5. Sensitivity, Specificity, and Predictive Value of Biomarkers

We evaluated the diagnostic potential of FINC and PMGE for atherothrombotic stroke, and of TPM1 for cardioembolic stroke (Table 2). FINC showed enough sensitivity (51.9%) and high specificity (85.2%), with a positive predictive value (PPV) of 77.8% and a negative predictive value (NPV) of 63.9%. PMGE had lower sensitivity (37.0%) but higher specificity (92.6%), with a PPV of 83.3% and an NPV of 59.5%. Additionally, TPM1 displayed a sensitivity of 40.7% and a specificity of 88.9%, with a PPV of 78.6% and an NPV of 60.0%. These results highlight the potential of these biomarkers in distinguishing individually between atherothrombotic and cardioembolic stroke cases.

The combination of these proteins in a biomarker panel, in cases in which any of the atherothrombotic markers (FINC or PMGE) was detected and the cardioembolic marker TPM1 was not detected, provides a sensitivity of 59.3% to correctly identify atherothrombotic etiology, and a specificity of 100% to exclude cardioembolic etiology, with a predictive value (PPV) of 100%, that is, with a 100% probability that the case is atherothrombotic. The negative predictive value (NPV) is 71.0%, indicating a 71.0% probability that cases without this combination are truly cardioembolic. Finally, considering the presence of TPM1 and absence of FINC and PMGE, this combination yields a sensitivity of 18.5% for detecting cardioembolic cases and a specificity of 100% for excluding atherothrombotic cases, with a 100% probability (PPV) that the case is cardioembolic, and a 55.1% probability (NPV) that cases without this combination are truly atherothrombotic.

## 3. Discussion

Identifying biomarkers to enhance diagnostic accuracy and help determine stroke etiology is necessary, as understanding the cause of stroke could be essential for both timely intervention and secondary stroke prevention [29]. Cardioembolic and atherothrombotic strokes require distinct therapeutic approaches to secondary prevention. Anticoagulant therapy is the treatment for cardioembolic stroke, while antiplatelet therapy is recommended for atherothrombotic or other non-embolic strokes [30]. Approximately 30% of stroke patients are initially classified as cryptogenic according to the TOAST criteria, although a specific cause is often identified later [31]. The biochemical pathways underlying these stroke mechanisms may differ. Therefore, we expect to find proteins linked to heart pathology contained in cardioembolic thrombi, while atherothrombotic thrombi may incorporate proteins related to the pathophysiology of atherosclerosis. Despite extensive research, no individual biomarker has yet demonstrated sufficient diagnostic and predictive accuracy for clinical use [32]. In this study, we present the discovery of three biomarkers that could serve as complementary tools to improve diagnostic accuracy.

Other studies have explored the composition of human clots. One small study identified 341 proteins common to all samples from four ischemic stroke patients [33]. Bioinformatic analyses revealed clusters of proteins associated with immune functions, cardiopathy, and vascular processes, as well as four proteins related to platelet function and clots, such as fibronectin 1, the 14-3-3 family, and TGFβ signaling. Although limited by sample size, these findings demonstrate the feasibility of proteomic studies in thrombi from ischemic stroke patients. Other proteomic studies identify and correlate different expressed proteins of the thrombi with ischemic stroke etiology. Dargazanli et al. [34] identified 438 different expressed proteins clustered in pathways related to metabolism, cell adhesion, and leukocyte activation, identifying three proteins, eIF2 subunit 3, Ras GTPase-activating-like protein IQGAP2, and coagulation factor XIII, with a significant ability to differentiate between stroke subtypes, correctly classifying cardioembolic (CE) and large artery atherosclerosis (LAA) stroke groups with 88% accuracy using mass spectrometry. Rossi et al. [35] identified 14 out of 1581 differentially expressed proteins involved in distinct pathways between LAA and CE stroke etiologies. Abbasi et al. [36] further demonstrated that platelet signaling and platelet–immune cell communication were predominant in CE compared to LAA thrombi. More recently, Lopez-Pedrera et al. [37] analyzed the protein composition of thrombi using a high-throughput mass spectrometry technique, identifying 580 proteins, and characterized a protein signature of atherothrombotic and cardioembolic strokes.

This research focused on the proteomic analysis of cardioembolic and atherothrombotic thrombi, identifying candidate proteins that distinguish between these two selected populations. The goal was to identify biomarkers that aid in stroke diagnosis and secondary stroke prevention, predict patient outcomes, and uncover new insights into the disease. Our proteomic study and statistical analysis identified three proteins, TPM1, FINC, and PMGE, which differ significantly between cardioembolic and atherothrombotic stroke groups. Our study is one of the largest conducted to date that analyzes thrombus composition; however, sample size needs to be expanded to broaden its potential applicability.

Tropomyosin-1 (TPM1) binds to actin filaments in muscle and non-muscle cells. In association with the troponin complex, TPM1 regulates calcium-dependent contraction in muscle cells. Calcium binding to troponin causes conformational changes that shift TPM1 along the actin filament, exposing myosin-binding sites and leading to sarcomere shortening and muscle contraction [38]. TPM1 is involved in abnormal contractions of cardiac muscle [39], and may contribute to cardioembolic stroke, highlighting its potential as a biomarker for muscle damage, similar to troponin C in diagnosing cardiac injury in ischemic stroke [40].

Fibronectin (FINC) is a dimeric glycoprotein found in the extracellular matrix. FINC is secreted by hepatocytes in response to vascular damage or inflammation, such as in atherosclerosis, and is incorporated into thrombi, where it plays roles in hemostasis, platelet aggregation and adhesion, tissue remodeling, and fibrinolysis. Each FINC dimer subunit contains an N-terminal domain that binds fibrin via factor XIIIa, stabilizing the thrombus [41]. FINC also contributes to tissue repair through its insoluble isoform and has been implicated in predicting hemorrhagic transformation and malignant ischemic stroke [42].

The enzyme 2,3-bisphosphoglycetate mutase (PMGE) participates in the secondary glycolytic route primarily active in mammalian erythrocytes. Under hypoxic conditions, PMGE activity in erythrocytes increases. PMGE catalyzes the conversion of 1,3-bisphosphoglycerate (1,3-BPG) into 2,3-bisphosphoglycerate (2,3-BPG), which binds to hemoglobin, decreasing its affinity for oxygen, stabilizing its deoxygenated form, and facilitating oxygen release to tissues [43]. During ischemic stroke, maintaining an adequate oxygen supply to the brain is critical for neuronal survival.

Our study reveals that TPM1 exhibits a sensitivity of 40.7% and a specificity of 85.2% for diagnosing cardioembolic stroke. In contrast, FINC and PMGE show specificities of 85.2% and 92.6% and sensitivities of 51.9% and 37.0%, respectively, for diagnosing atherothrombotic stroke when assessed individually. This is the first time TPM1 has been identified as a potential biomarker for the cardioembolic stroke subtype, while FINC and PMGE have been proposed as potential biomarkers for atherothrombotic stroke. Furthermore, we explored whether combining these three proteins could improve diagnostic accuracy. For cardioembolic stroke, a positive TPM1 result along with negative FINC and PMGE results yielded 100% specificity and predictive value. Similarly, for atherothrombotic stroke, positive results for FINC and PMGE with a negative TPM1 result also achieved 100% specificity and predictive value. These findings show that analyzing combinations of TPM1, FINC, and PMGE could provide a highly reliable diagnostic tool for distinguishing between cardioembolic and atherothrombotic stroke subtypes. Thus, it is noteworthy that these three biomarkers could serve as complementary tools to improve diagnostic accuracy, which could be particularly useful in determining the cause of disease in stroke patients initially classified as cryptogenic.

Predicting outcome is important to guide treatment and communicate with patients and their families regarding the expected effects of a stroke. Biomarkers offer the potential to predict prognosis in stroke, including patient response to treatment, development of complications, and long-term functional outcomes. In our study of biomarker correlations with clinical variables, we propose a dual role for FINC as a cell adhesion protein involved in tissue regeneration and stroke pathology. Elevated levels of FINC peptides were detected in cases with less brain injury, both at discharge and after three months. However, elevated FINC levels were also associated with more severe brain damage following stroke. This aligns with other studies linking FINC to hemorrhagic transformation, cerebral edema, and poor prognosis [43]. FINC, located in the endothelium and substrate for matrix metalloproteinase-9 (MMP-9), is involved in blood–brain barrier (BBB) disruption, with high levels linked to parenchymal hematoma after rtPA treatment [44]. Similarly, higher levels of PMGE have been observed in younger patients. Moreover, we also found that PMGE levels decrease with the recanalization time, possibly due to the detachment of this protein from the thrombus. In contrast to FINC, which is structurally part of the thrombus and increases over time, PMGE exhibits the opposite trend. Finally, in the case of TPM1, we found a correlation between its levels and baseline NIHSS scores, indicating that TPM1 may serve as a marker of neurological deficit, particularly in cases of cardioembolic stroke.

Regarding the proteomics approach used, we performed a proteomic analysis based on in-gel MALDI-TOF MS, with less sensitivity than LC-MS/MS in detecting low-abundance proteins, because we focused the proteomic study on the main components of the thrombus—more characteristic of a thrombus collection—and less on the identification of particular proteins of a thrombus, and because this technique provides robust identifications, avoids false positive identifications, and distinguishes different proteoforms.

In this study, three biomarkers—TPM1, FINC, and PMGE—have been identified by means of a differential proteomic analysis comparing thrombi of cardioembolic and atherothrombotic etiology. The combination of these proteomics-based findings with other imaging biomarkers based on quantitative magnetic resonance imaging (MRI) analysis using emerging techniques such as quantitative susceptibility mapping (QSM) [45], could open up a new field of research by integrating imaging and omics biomarkers, which could represent a qualitative advance in the study and prognosis of ischemic stroke.

In summary, this work presents the discovery of a biomarker panel of three proteins, TPM1, FINC, and PMGE, which differentiate cardioembolic from atherothrombotic stroke. Combined biomarkers showed 100% specificity for both stroke subtypes, underscoring the potential of these proteins as complementary tools for precise diagnosis of ischemic stroke etiology, which is especially useful in cases classified as cryptogenic in origin. Timely diagnosis and intervention are critical in stroke management, and biomarkers can play a key role in improving diagnostic accuracy and informing prognosis. Although these biomarkers should be further analyzed in prospective multicenter studies to demonstrate their usefulness, they may be promising diagnostic tools to confirm the etiology of ischemic stroke, including cases of cryptogenic etiology.

## 4. Materials and Methods

### 4.1. Subjects

Thrombi from stroke patients for this study were randomly selected from individuals with LVO, either cardioembolic or atherothrombotic, who underwent mechanical thrombectomy. Patients were recruited from the Stroke Unit of the Neurology Department at Ramón y Cajal University Hospital between September 2019 and December 2023, and strokes were classified according to the TOAST criteria [11]. Thrombi were extracted via mechanical thrombectomy, following the standard procedure established at Hospital Universitario Ramón y Cajal, in accordance with the protocol recommendations for endovascular treatment of acute ischemic stroke from the Stroke Care Plan of the Region of Madrid, performed by experienced interventional neuroradiologists.

A sample size analysis (https://es.surveymonkey.com/mp/sample-size-calculator/, accessed on 18 December 2023) was performed to determine the necessary sample size representative of the thrombus collection from stroke patients (347 cases). Using a significance level of 0.05 and a margin of error of 15%, the requirement was at least 40 cases, i.e., 20 cases per group. Accordingly, thrombi from 27 patients in each of the cardioembolic and atherothrombotic stroke groups were analyzed. Data on stroke etiology, patient status, and demographics were collected from the database for these patients, ensuring anonymity and analyzed blindly. Data from patients’ thrombi were independently analyzed and patient and treatment information was kept blinded throughout the study.

Informed consent was obtained from all participants, and the study was approved by the Research Ethics Committee of Ramón y Cajal University Hospital (Madrid, Spain), in accordance with the Declaration of Helsinki.

### 4.2. Thrombi Processing

Thrombi from stroke patients obtained via mechanical thrombectomy were initially rinsed and preserved in saline solution at 4 °C until transport to the laboratory. Thrombi underwent an additional wash with phosphate-buffered saline (PBS) at 4 °C. The thrombi were then dissected into consecutive, uniform fragments from head to tail, with a consistent weight, preferably around 20 mg per fragment. Each fragment was accurately weighted, its observed characteristics recorded, and stored in a laboratory freezer at −80 °C for later use.

### 4.3. Protein Extraction

Protein extraction was performed by manual homogenization of the thrombus head fragment (5–25 mg) (1:5, *w*/*v*) in lysis buffer A (20 mM tris-HCl, pH 7.6, 140 mM KCl, 2.5 mM magnesium acetate, 1 mM EDTA, 2 mM EGTA, 2 mM benzamidine, 20 mM β-glycerophosphate, 2 mM sodium molibdate, 0.2 mM sodium ortovanadate, and 10 µg/mL pestatin A, leupeptin and antipain), 1% Triton X-100, 0.1% SDS, and 0.1% sodium deoxycholate, using a small plastic homogenizer. After homogenization, samples were centrifuged at 100,000× *g* for 1 h in a TL-100 Ultracentrifuge (Beckman Coulter Life Sci., Barcelona, Spain). The supernatant was collected, aliquoted, and stored at −80 °C for further analysis. Protein content was quantified using the Bradford (Bio-Rad, Madrid, Spain) assay.

### 4.4. Gel Electrophoresis

Protein extracts (100 μg) were separated and resolved using denaturing sodium dodecyl sulphate–polyacrylamide gel electrophoresis (SDS-PAGE) with a 12% acrylamide gel with 2.6% cross-linking. A low-molecular-weight calibration kit (Cytiva, formerly GE Healthcare, Barcelona, Spain) was used as a molecular mass standard. Proteins were stained with Coomassie brilliant blue R-250 (Bio-Rad) and then destained with a solution of 20% ethanol and 5% acetic acid (*v*/*v*). The gels were then preserved in 10% ethanol until scanning and protein extraction for further identification by mass spectrometry (MS).

### 4.5. In-Gel Protein Digestion and Protein Identification by Mass Spectrometry

**Figure 4:**
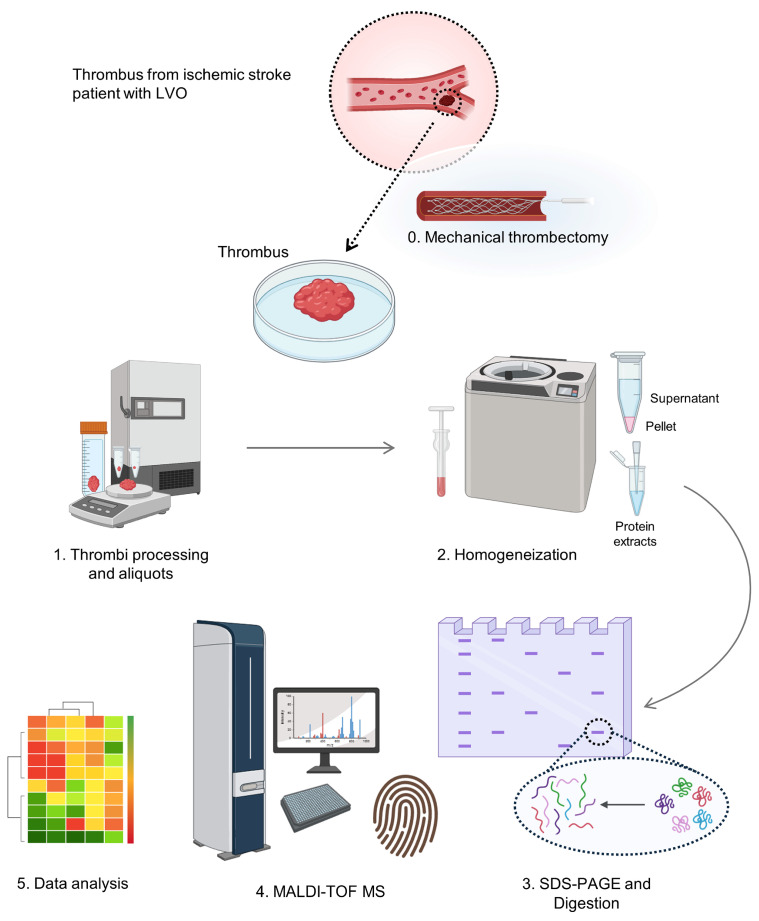
Clinical proteomics workflow performed for the identification of the protein composition of a thrombus from a patient with ischemic stroke.

Gel bands were manually excised from the Coomassie blue-stained gels for tryptic digestion with modified porcine trypsin (Promega, Madrid, Spain). The digestion protocol followed the method of Shevchenko et al. [46], with minor modification [47]. After digestion, peptide mass fingerprinting (PMF) was analyzed by matrix-assisted laser desorption/ionization time-of-flight mass spectrometry (MALDI-TOF MS) (UltrafleXtreme, Bruker-Daltonics, Bremen, Germany) for protein identification, as detailed in earlier studies [48]. Workflow of this study is schematized in Figure 4.

### 4.6. Quantification of Peptides by Mass Spectrometry

After in-gel protein digestion, angiotensin-II peptide (40 fmol) was added to the collected tryptic peptides. Peptides obtained from the digestion and the angiotensin-II peptide were analyzed by MALDI-TOF MS, and peptide intensities in the MS spectra were quantified relative to the angiotensin-II peptide intensity as a ratio of peptide intensity to angiotensin-II intensity, as described in earlier studies [49].

### 4.7. Data Collection and Statistical Analysis

Data were collected and analyzed blindly. Statistical analysis was conducted to compare and identify differences between groups using Excel spreadsheet (Microsoft) and Prism 5.0 statistical software package (GraphPad Software). Data were expressed as mean ± standard deviation (SD) for continuous variables, or in percentages (%) for categorical variables. Comparisons between groups were made using the Student’s *t*-test for continuous variables and Fisher’s exact test for categorical variables. The receiver operating characteristic (ROC) curves and area under the curve (AUC) were obtained with the software package. Spearman’s rank correlation coefficient was used to assess linear correlations.

## Figures and Tables

**Table 1:** Clinical characteristics of the patients according to stroke etiology.

	**Cardioembolic Etiology** (range/percentage)	**Atherothrombotic Etiology** (range/percentage)	***p*-Value; Result ^1^**
**Demographic data**			
Age (years)	78 (41–90)	64 (35–89)	0.002; **
Gender, female	15 (55.6%)	8 (29.6%)	0.098; ns
**Risk Factors**			
Hypertension	21 (77.8%)	15 (55.6%)	0.148; ns
Diabetes mellitus	6 (22.2%)	8 (29.6%)	0.757; ns
Hyperlipidemia	21 (77.8%)	22 (81.5%)	0.999; ns
Smoking habit	2 (7.4%)	12 (44.4%)	0.004; **
Drinking habit	1 (3.7%)	4 (14.8%)	0.351; ns
Atrial fibrillation	24 (88.9%)	0 (0.0%)	0.000; ****
Ischemic heart disease	3 (11.1%)	4 (14.8%)	0.999; ns
Previous stroke	4 (14.8%)	5 (18.5%)	0.999; ns
**LVO localization**			
MCA	15 (55.6%)	6 (22.2%)	0.024; *
Basilar artery	4 (14.8%)	4 (14.8%)	0.999; ns
MCA + carotid artery	8 (29.6%)	17 (63.0%)	0.028; *
**In-hospital work-up**			
Secondary transfer	10 (37.0%)	13 (48.2%)	0.583; ns
Onset-to-recanalization time (minutes) ± SD	112.8 ± 13.0	143.3 ± 27.8	0.331; ns
Pre-stroke mRS	0 (0–3)	0 (0–3)	0.999; ns
Baseline NIHSS	18 (9–26)	15 (3–23)	0.004; **
NIHSS at discharge	4 (0–26)	4 (0–30)	0.460; ns
90-day mRS	2 (0–6)	2 (0–6)	0.576; ns
Death in hospital	2 (7.4%)	5 (19.2%)	0.250; ns
Stent	0 (0.0%)	21 (77.8%)	0.000; ****
rtPA administration	11 (40.7%)	17 (63.0%)	0.173; ns

Abbreviations used: LVO: large vessel occlusion; MCA: middle cerebral artery; mRS: modified Rankin Scale; NIHSS: National Institute of Health Stroke Scale; rtPA: recombinant tissue plasminogen activator. ^1^ ns, not significant. *, *p* < 0.05; **, *p* < 0.01; and ****, *p* < 0.0001; by Student’s *t*-test or Fisher’s exact test.

**Table 2:** Sensitivity and specificity of biomarkers and combination in biomarker panels for the diagnosis of cardioembolic or atherothrombotic ischemic stroke.

	Sensitivity	Specificity	PPV ^1^	NPV ^2^
Biomarker panel associated with atherothrombotic thrombi				
FINC ^+^	0.52	0.85	0.78	0.64
PMGE ^+^	0.37	0.93	0.83	0.60
FINC ^+^ or PMGE ^+^, TPM1 ^−^	0.59	1.0	1.0	0.71
Biomarker panel associated with cardioembolic thrombi				
TPM1 ^+^	0.41	0.89	0.79	0.60
FINC ^−^, PMGE ^−^, TPM1 ^+^	0.19	1.0	1.0	0.55

^1^ PPV, Positive predictive value. ^2^ NPV, Negative predictive value.

## Data Availability

The raw data supporting the conclusions of this article will be made available by the authors on reques.

## Supplementary Materials

The following supporting information can be downloaded at: https://www.mdpi.com/article/10.3390/ijms26178333/s1.
