# Supplementary material for: Identification of Differential Proteins in Thrombi of Cardioembolic and Atherothrombotic Etiology in Patients with Ischemic Stroke
Source: Int J Mol Sci. 2025 Aug 28;26(17):8333. doi: 10.3390/ijms26178333 (PMC12428142; doi:10.3390/ijms26178333)
Supplement: Supplementary file 1 [file ijms-26-08333-s001.zip › ijms-3822019-supplementary.pdf]

Table S1. Complete list of proteins identified by mass spectrometry MALDI-TOF

Protein ID /Abbreviation	Access no. Uniprot [a]	Gene name	Molecular mass (Da)	Score [b]	Peptide match /total	Coverage (%)	Cardioembolic	Aterotrombotic	Fisher's test	Chi-square	Significance
Nesprin-1 /SYNE1	Q8NF91	SYNE1	1017127	80	68/90	5			0.491	0.150	ns
Microtubule-actin cross-linking factor 1, isoforms 1/2/3/5 /MACF1	Q9UPN3	MACF1	843033	60	58/98	7			1.000	0.313	ns
Nesprin-2 /SYNE2	Q8WXH0	SYNE2	801817	59	54/83	5			1.000	0.313	ns
Fibrous sheath-interacting protein 2 /FSIP2	Q5CZC0	FSIP2	785878	62	53/82	6			1.000	0.313	ns
Plectin /PLEC	Q15149	PLEC	533462	69	48/96	9			0.491	0.150	ns
Sacsin /SACS	Q9NZJ4	SACS	526497	62	42/96	8			1.000	1.000	ns
Dynein heavy chain 8, axonemal /DYH8	Q96JB1	DNAH8	517984	62	31/56	5			1.000	0.313	ns
Dynein heavy chain 10, axonemal /DYH10	Q8IVF4	DNAH10	517705	62	51/96	8			1.000	0.553	ns
Apolipoprotein B-100 /APOB	P04114	APOB	516651	59	42/96	7			0.491	0.150	ns
Ankyrin-3 /ANK3	Q12955	ANK3	482394	62	45/90	9			0.491	0.150	ns
Dynein axonemal heavy chain 6 /DYH6	Q9C0G6	DNAH6	479671	60	41/91	7			1.000	1.000	ns
A-kinase anchor protein 9 /AKAP9	Q99996	AKAP9	454989	61	45/96	9			1.000	0.313	ns
Spectrin beta chain, non-erythrocytic 5 /SPTN5	Q9NRC6	SPTBN5	419230	69	32/69	6			1.000	1.000	ns
Utrophin /UTRO	P46939	UTRN	396444	60	41/90	10			1.000	0.313	ns
Xin actin-binding repeat-containing protein 2 /XIRP2	A4UGR9	XIRP2	383888	67	42/97	10			1.000	0.313	ns
Golgin subfamily B member 1 /GOGB1	Q14789	GOLGB1	377215	77	44/91	11			0.610	0.299	ns
Centromere protein F /CENPF	P49454	CENPF	360442	83	44/84	9			1.000	1.000	ns
WD repeat-containing protein 87 /WDR87	Q6ZQQ6	WDR87	335256	61	36/93	9			1.000	0.313	ns
Nipped-B-like protein /NIBLP	Q6KC79	NIPBL	317907	63	38/98	10			1.000	0.313	ns
Centromere-associated protein E /CENPE	Q02224	CENPE	317588	77	48/96	15			1.000	0.639	ns
Serine/arginine repetitive matrix protein 2 /SRRM2	Q9UQ35	SRRM2	300179	68	42/96	12			0.491	0.150	ns
ATP-binding cassette sub-family A member 12 /ABCAC	Q86UK0	ABCA12	295387	56	27/69	8			1.000	0.313	ns
eIF-2-alpha kinase activator GCN1 /GCN1	Q92616	GCN1	294919	57	25/86	10			1.000	0.313	ns
Centrosomal protein of 290 kDa /CE290	O15078	CEP290	290892	70	47/98	14			0.491	0.150	ns
Zinc finger protein 462 /ZN462	Q96JM2	ZNF462	289467	62	30/87	9			1.000	0.313	ns
Spectrin alpha chain, non-erythrocytic 1 /SPTN1	Q13813	SPTAN1	285163	93	28/48	8			0.420	0.224	ns
Voltage-dependent P/Q-type calcium channel subunit alpha-1A /CAC1A	O00555	CACNA1A	283981	61	13/24	3			1.000	0.313	ns
Filamin-A /FLNA	P21333	FLNA	283301	207	47/97	18			0.276	0.174	ns
Centrosome-associated protein CEP250 /CP250	Q9BV73	CEP250	281880	59	34/95	12			1.000	1.000	ns
Spectrin alpha chain, erythrocytic /SPTA1	P02549	SPTA1	281039	109	45/96	15			0.704	0.444	ns
**Fibronectin /FINC**	P02751	FN1	275742	103	20/97	7			0.008	0.004	**p < 0.01**
Talin-2 /TLN2	Q9Y4G6	TLN2	273781	68	21/71	8			1.000	0.313	ns
Talin-1 /TLN1	Q9Y490	TLN1	271766	140	40/99	17			1.000	1.000	ns
Nucleoprotein TPR /TPR	P12270	TPR	267530	91	41/87	13			1.000	0.685	ns
Golgin subfamily A member 4 /GOGA4	Q13439	GOLGA4	261892	67	43/99	15			1.000	1.000	ns
Protein dopey-2 /DOP2	Q9Y3R5	DOP1B	260348	65	29/97	11			1.000	0.313	ns
Kinetochore-associated protein 1 /KNTC1	P50748	KNTC1	253211	61	28/97	9			1.000	0.313	ns
Activating signal cointegrator 1 complex subunit 3 /ASCC3	Q8N3C0	ASCC3	252898	58	15/33	5			1.000	0.313	ns
Spectrin beta chain, erythrocytic /SPTB1	P11277	SPTB	247171	183	49/99	21			1.000	0.736	ns
1-phosphatidylinositol 3-phosphate 5-kinase /FYV1	Q9Y2I7	PIKFYVE	239609	64	22/84	9			1.000	0.313	ns
Unconventional myosin-XVIIIa /MY18A	Q92614	MYO18A	234168	69	35/90	12			0.491	0.150	ns
Citron Rho-interacting kinase /CTRO	O14578	CIT	233339	82	36/96	14			1.000	0.553	ns
Envoplakin /EVPL	Q92817	EVPL	232774	62	30/98	12			0.491	0.150	ns
Protein Daple /DAPLE	Q9P219	CCDC88C	229231	64	26/69	8			1.000	0.313	ns
Rootletin /CROCC	Q5TZA2	CROCC	228787	69	36/98	15			0.544	0.362	ns
SMC hinge domain-containing protein 1 /SMHD1	A6NHR9	SMCHD1	227942	57	12/49	4			1.000	0.313	ns
Myosin-9 /MYH9	P35579	MYH9	227646	219	50/99	26			0.785	0.584	ns
CASP8-associated protein 2 /C8AP2	Q9UKL3	CASP8AP2	224858	66	37/95	12			1.000	1.000	ns
Myosin-13 /MYH13	Q9UKX3	MYH13	224605	57	24/56	12			1.000	0.313	ns
Exophilin-5 /EXPH5	Q8NEV8	EXPH5	223751	68	31/95	11			1.000	0.313	ns
Sickle tail protein homolog /SKT	Q5T5P2	KIAA1217	214610	66	31/95	13			1.000	0.553	ns
Chromodomain-helicase-DNA-binding protein 2 /CHD2	O14647	CHD2	212183	58	33/87	11			1.000	0.313	ns
Kinesin-like protein KIF20B /KI20B	Q96Q89	KIF20B	211868	58	24/68	10			1.000	0.313	ns
C-myc promoter-binding protein /MYCPP	Q7Z401	DENND4A	211791	57	23/86	9			1.000	0.313	ns
Fibronectin type III domain-containing protein 1 /FNDC1	Q4ZHG4	FNDC1	205889	59	26/98	9			1.000	0.313	ns
Periplakin /PEPL	O60437	PPL	205193	65	33/98	14			1.000	0.313	ns
E3 ubiquitin-protein ligase RBBP6 /RBBP6	Q7Z6E9	RBBP6	202354	68	28/97	13			0.236	0.075	ns
Nebulin-related-anchoring protein /NRAP	Q86VF7	NRAP	197920	68	35/95	15			1.000	0.553	ns
Ankyrin repeat domain-containing protein 26 /ANR26	Q9UPS8	ANKRD26	197487	57	28/97	13			1.000	0.313	ns
GRIP and coiled-coil domain-containing protein 2 /GCC2	Q8IWJ2	GCC2	196872	64	13/42	9			1.000	0.313	ns
Protein polybromo-1 /PB1	Q86U86	PBRM1	194080	64	32/96	14			1.000	0.313	ns

Maestro heat-like repeat-containing protein family member 2A /MRO2A	A6NES4	MROH2A	191491	60	24/98	14			1.000	0.313	ns
TOG array regulator of axonemal microtubules protein 1 /TGRM1	Q9Y4F4	TOGARAM1	190611	59	8/12	6			1.000	0.313	ns
Myomesin-1 /MYOM1	P52179	MYOM1	188992	58	9/35	6			1.000	0.313	ns
Kinesin-like protein KIF14 /KIF14	Q15058	KIF14	187743	61	31/88	13			1.000	1.000	ns
RB1-inducible coiled-coil protein 1 /RBCC1	Q8TDY2	RB1CC1	185085	78	32/96	16			1.000	0.313	ns
DNA topoisomerase 2-beta /TOP2B	Q02880	TOP2B	184122	70	29/97	16			0.704	0.444	ns
Cortactin-binding protein 2 /CTTB2	Q8WZ74	CTTNBP2	183332	62	24/97	13			1.000	0.313	ns
DNA topoisomerase 2-alpha /TOP2A	P11388	TOP2A	175017	68	23/96	12			0.610	0.299	ns
Synemin /SYNEM	O15061	SYNM	173105	57	25/91	10			1.000	0.313	ns
Centrosomal protein of 170 kDa protein B /C170B	Q9Y4F5	CEP170B	172154	58	18/78	13			1.000	1.000	ns
Synaptojanin-2 /SYNJ2	O15056	SYNJ2	166575	56	16/62	7			1.000	0.313	ns
Myomesin-2 /MYOM2	P54296	MYOM2	166332	66	21/79	11			1.000	0.313	ns
Probable ATP-dependent DNA helicase HFM1 /HFM1	A2PYH4	HFM1	163705	65	13/71	8			1.000	0.313	ns
Uveal autoantigen with coiled-coil domains and ankyrin repeats /UACA	Q9BZF9	UACA	163545	68	29/69	13			1.000	0.553	ns
Early endosome antigen 1 /EEA1	Q15075	EEA1	163337	74	32/96	18			1.000	0.553	ns
FYVE, RhoGEF and PH domain-containing protein 6 /FGD6	Q6ZV73	FGD6	162596	71	26/96	13			1.000	1.000	ns
Centrosomal protein of 162 kDa /CE162	Q5TB80	CEP162	162356	58	19/53	11			1.000	0.313	ns
Centlein /CNTLN	Q9NXG0	CNTLN	162099	60	28/97	14			1.000	0.313	ns
3'-5' RNA helicase YTHDC2 /YTDC2	Q9H6S0	YTHDC2	161573	61	12/56	8			1.000	0.313	ns
Kinesin-like protein KIF27 /KIF27	Q86VH2	KIF27	161324	61	27/97	18			0.491	0.150	ns
Regulating synaptic membrane exocytosis protein 2 /RIMS2	Q9UQ26	RIMS2	161216	64	22/72	11			1.000	0.313	ns
Rho-associated protein kinase 1 /ROCK1	Q13464	ROCK1	159102	64	26/77	15			1.000	0.313	ns
Spermatogenesis-associated protein 31E1 /S31E1	Q6ZUB1	SPATA31E1	158862	71	25/91	13			1.000	0.313	ns
Regulator of G-protein signaling 12 /RGS12	O14924	RGS12	157800	65	13/34	6			1.000	0.313	ns
Coiled-coil domain-containing protein 7 /CCDC7	Q96M83	CCDC7	157580	60	19/65	12			0.236	0.075	ns
DNA repair protein RAD50 /RAD50	Q92878	RAD50	154823	80	33/90	18			1.000	0.313	ns
Centromere protein J /CENPJ	Q9HC77	CENPJ	154103	58	25/90	17			1.000	0.313	ns
Coiled-coil domain-containing protein 171 /CC171	Q6TFL3	CCDC171	154083	57	22/62	11			1.000	0.313	ns
Multidrug resistance-associated protein 9 /MRP9	Q96J65	ABCC12	153625	56	13/39	6			1.000	0.313	ns
Ribosome-binding protein 1 /RRBP1	Q9P2E9	RRBP1	152764	56	24/71	13			1.000	0.313	ns
Kinesin-like protein KIF16B /KI16B	Q96L93	KIF16B	152488	72	24/75	15			0.491	0.150	ns
Sister chromatid cohesion protein PDS5 homolog A /PDS5A	Q29RF7	PDS5A	152274	66	25/93	15			1.000	0.313	ns
Kinesin-like protein KIF7 /KIF7	Q2M1P5	KIF7	151407	57	20/57	10			1.000	0.313	ns
Microtubule-associated tumor suppressor candidate 2 /MTUS2	Q5JR59	MTUS2	150901	62	20/80	10			1.000	0.639	ns
Fanconi anemia group I protein /FANCI	Q9NVI1	FANCI	150769	68	9/58	6			1.000	0.313	ns
Protein SFI1 homolog /SFI1	A8K8P3	SFI1	148939	76	30/98	18			1.000	0.313	ns
Ubiquitin carboxyl-terminal hydrolase 31 /UBP31	Q70CQ4	USP31	148328	56	13/56	12			1.000	0.313	ns
SH3 domain and tetratricopeptide repeat-containing protein 2 /S3TC2	Q8TF17	SH3TC2	146681	65	17/64	10			1.000	0.313	ns
Cilia- and flagella-associated protein 57 /CFA57	Q96MR6	CFAP57	145839	58	9/29	7			1.000	0.313	ns
Tubulin polyglutamylase TTLL5 /TTLL5	Q6EMB2	TTLL5	144457	64	20/98	13			1.000	0.313	ns
Putative coiled-coil domain-containing protein 144C /C144C	Q8IYA2	CCDC144CP	144106	62	10/18	8			0.491	0.150	ns
Structural maintenance of chromosomes protein 1A /SMC1A	Q14683	SMC1A	143771	59	26/96	19			1.000	0.313	ns
Zinc finger transcription factor Trps1 /TRPS1	Q9UHF7	TRPS1	143656	57	10/35	7			1.000	0.313	ns
AP2-interacting clathrin-endocytosis protein /K1107	Q9UPP5	KIAA1107	142315	57	12/33	7			1.000	0.313	ns
Ras-specific guanine nucleotide-releasing factor 2 /RGRF2	O14827	RASGRF2	142158	58	12/47	7			1.000	0.313	ns
Chromosome-associated kinesin KIF4A /KIF4A	O95239	KIF4A	141390	61	17/42	8			1.000	0.313	ns
Liprin-alpha-1 /LIPA1	Q13136	PPFIA1	136265	56	27/97	16			1.000	0.313	ns
Liprin-alpha-4 /LIPA4	O75335	PPFIA4	134948	65	14/41	6			1.000	0.313	ns
Protein WWC2 /WWC2	Q6AWC2	WWC2	134835	64	22/89	17			1.000	1.000	ns
Centrosomal protein of 135 /CP135	Q66GS9	CEP135	133864	71	23/74	17			1.000	1.000	ns
Thrombospondin-1 /TSP1	P07996	THBS1	133291	123	26/99	23			1.000	0.313	ns
Protein FAM184A /F184A	Q8NB25	FAM184A	133111	63	16/74	13			1.000	0.313	ns
Unconventional myosin-Ib /MYO1B	O43795	MYO1B	132928	57	26/95	14			1.000	0.313	ns
AF4/FMR2 family member 1 /AFF1	P51825	AFF1	132139	59	8/27	5			1.000	0.313	ns
Filamin A-interacting protein 1-like /FIL1L	Q4L180	FILIP1L	130928	78	28/98	21			0.236	0.075	ns
Retinoblastoma-like protein 2 /RBL2	Q08999	RBL2	129711	58	17/78	13			1.000	0.313	ns
Coiled-coil domain-containing protein 150 /CC150	Q8NCX0	CCDC150	129536	59	8/14	7			1.000	0.313	ns
DNA helicase MCM9 /MCM9	Q9NXL9	MCM9	128715	65	15/97	11			1.000	0.313	ns
Centrosomal protein of 128 /CE128	Q6ZU80	CEP128	128565	58	16/51	11			1.000	0.313	ns
Integrin alpha-M /ITAM	P11215	ITGAM	128410	64	17/97	12			1.000	0.553	ns
ELKS/Rab6-interacting/CAST family member /RB6I2	Q8IUD2	ERC1	128236	65	19/52	13			0.610	0.299	ns
Elongation factor-like GTPase 1 /EFL1	Q7Z2Z2	EFL1	126661	67	18/77	12			1.000	0.313	ns
Coiled-coil domain-containing protein 73 /CCD73	Q6ZRK6	CCDC73	125046	57	15/53	12			1.000	0.313	ns
Vinculin /VINC	P18206	VCL	124292	176	36/97	31			0.766	0.551	ns
Protein WWC3 /WWC3	Q9ULE0	WWC3	123741	58	17/80	15			1.000	0.313	ns
TATA element modulatory factor /TMF1	P82094	TMF1	123280	62	12/50	8			1.000	0.313	ns
POTE ankyrin domain family member F /POTEF	A5A3E0	POTEF	123020	58	21/98	15			1.000	0.313	ns
POTE ankyrin domain family member E /POTEE	Q6S8J3	POTEE	122882	56	14/70	11			1.000	0.553	ns
Formin-like protein 1 /FMNL1	O95466	FMNL1	122518	101	25/95	17			1.000	0.761	ns
Protein FAM184B /F184B	Q9ULE4	FAM184B	121882	61	15/54	16			1.000	0.313	ns
Zinc finger protein 445 /ZN445	P59923	ZNF445	121282	63	24/89	16			1.000	0.313	ns

Pre-mRNA-splicing factor ATP-dependent RNA helicase DHX16 /DHX16	O60231	DHX16	119874	73	16/77	12			0.610	0.299	ns
Disks large-associated protein 2 /DLGP2	Q9P1A6	DLGAP2	118915	60	20/98	15			1.000	0.313	ns
Caspase recruitment domain-containing protein 10 /CAR10	Q9BWT7	CARD10	117342	58	16/69	12			1.000	0.313	ns
Serine/threonine-protein kinase 31 /STK31	Q9BXU1	STK31	116647	61	19/97	15			1.000	0.313	ns
Probable E3 ubiquitin-protein ligase HERC6 /HERC6	Q8IVU3	HERC6	116535	58	9/40	7			1.000	0.313	ns
CAP-Gly domain-containing linker protein 2 /CLIP2	Q9UDT6	CLIP2	116223	74	20/99	18			0.728	0.484	ns
Immunoglobulin superfamily member 2 /IGSF2	Q93033	CD101	116063	60	12/61	14			1.000	0.313	ns
Protein phosphatase 1 regulatory subunit 12A /MYPT1	O14974	PPP1R12A	115610	56	16/83	12			1.000	0.313	ns
Integrin alpha-IIb /ITA2B	P08514	ITGA2B	114446	208	32/97	31			0.420	0.224	ns
Potassium voltage-gated channel subfamily H member 5 /KCNH5	Q8NCM2	KCNH5	112946	62	9/38	11			1.000	0.313	ns
Serine/threonine-protein kinase 10 /STK10	O94804	STK10	112749	67	25/91	16			0.236	0.075	ns
PDZ domain-containing protein 7 /PDZD7	Q9H5P4	PDZD7	112196	72	20/81	13			0.491	0.150	ns
TOG array regulator of axonemal microtubules protein 2 /TGRM2	Q6ZUX3	TOGARAM2	111996	62	21/95	19			1.000	0.313	ns
Inositol polyphosphate-4-phosphatase type I A /INP4A	Q96PE3	INPP4A	111539	62	8/31	9			1.000	0.313	ns
Protein bicaudal D homolog 1 /BICD1	Q96G01	BICD1	111252	73	25/97	21			1.000	0.313	ns
VPS35 endosomal protein-sorting factor-like /VP35L	Q7Z3J2	VPS35L	110860	64	12/49	11			1.000	0.313	ns
ERC protein 2 /ERC2	O15083	ERC2	110661	68	19/62	13			1.000	1.000	ns
TBC1 domain family member 2B /TBD2B	Q9UPU7	TBC1D2B	110496	98	8/27	6			1.000	1.000	ns
Kinesin-1 heavy chain /KINH	P33176	KIF5B	110358	60	14/52	15			1.000	0.313	ns
Bromodomain testis-specific protein /BRDT	Q58F21	BRDT	108514	57	12/62	12			1.000	0.313	ns
Coiled-coil domain-containing protein 14 /CCD14	Q49A88	CCDC14	107206	67	16/73	13			1.000	0.313	ns
Angiomotin-like protein 1 /AMOL1	Q8IY63	AMOTL1	106850	70	13/55	12			1.000	0.313	ns
Bcl-2-associated transcription factor 1 /BCLF1	Q9NYF8	BCLAF1	106173	65	18/53	15			1.000	0.313	ns
Rho GTPase-activating protein 4 /RHG04	P98171	ARHGAP4	105816	66	20/95	15			1.000	0.313	ns
Inositol polyphosphate 5-phosphatase OCRL /OCRL	Q01968	OCRL	105392	64	11/46	10			1.000	0.313	ns
Alpha-actinin-4 /ACTN4	O43707	ACTN4	105245	61	12/51	12			1.000	0.553	ns
Ankyrin repeat and LEM domain-containing protein 2 /ANKL2	Q86XL3	ANKLE2	104905	65	19/80	14			0.491	0.150	ns
Coiled-coil and C2 domain-containing protein 1A /C2D1A	Q6P1N0	CC2D1A	104397	65	19/75	12			1.000	0.313	ns
Protein O-GlcNAcase /OGA	O60502	OGA	103932	74	12/60	9			1.000	0.313	ns
Alpha-actinin-1 /ACTN1	P12814	ACTN1	103563	199	35/97	34			0.293	0.161	ns
Kinesin-like protein KIF18A /KI18A	Q8NI77	KIF18A	103414	56	15/72	14			1.000	0.313	ns
Protein CIP2A /CIP2A	Q8TCG1	CIP2A	103318	56	20/98	19			0.491	0.150	ns
G protein-regulated inducer of neurite outgrowth 1 /GRIN1	Q7Z2K8	GPRIN1	103134	67	19/80	16			0.491	0.150	ns
Scaffold attachment factor B1 /SAFB1	Q15424	SAFB	103036	57	10/20	7			1.000	0.313	ns
DNA replication licensing factor MCM2 /MCM2	P49736	MCM2	102516	73	19/97	18			1.000	0.553	ns
Band 3 anion transport protein /B3AT	P02730	SLC4A1	102013	108	13/28	20			0.669	0.387	ns
Smoothelin /SMNT	P53814	SMTN	99511	60	11/84	14			1.000	0.313	ns
Rho GTPase-activating protein 42 /RHG42	A6NI28	ARHGAP42	99248	63	8/28	7			1.000	1.000	ns
LINE-1 type transposase domain-containing protein 1 /LITD1	Q5T7N2	L1TD1	99245	71	16/50	18			1.000	0.313	ns
Cytoplasmic aconitate hydratase /ACOC	P21399	ACO1	98850	63	8/48	11			1.000	0.313	ns
Janus kinase and microtubule-interacting protein 3 /JKIP3	Q5VZ66	JAKMIP3	98640	66	14/69	11			1.000	0.313	ns
Dynamin-1 /DYN1	Q05193	DNM1	97746	60	14/69	17			0.236	0.075	ns
Glycogen phosphorylase, muscle form /PYGM	P11217	PYGM	97487	68	17/99	17			1.000	0.313	ns
GRIP1-associated protein 1 /RAP1	Q4V328	GRIPAP1	96289	69	21/88	19			0.669	0.387	ns
Fibrinogen alpha chain /FIBA	P02671	FGA	95656	238	41/98	35			0.586	0.413	ns
Vacuolar protein sorting-associated protein 53 homolog /VPS53	Q5VIR6	VPS53	95200	65	7/27	4			1.000	0.313	ns
Coiled-coil and C2 domain-containing protein 1B /C2D1B	Q5T0F9	CC2D1B	94394	63	19/96	17			1.000	0.313	ns
Coiled-coil domain-containing protein 175 /CC175	P0C221	CCDC175	94023	58	15/48	14			1.000	0.313	ns
Protein bicaudal D homolog /BICD2	Q8TD16	BICD2	93704	63	14/73	14			1.000	1.000	ns
MAP7 domain-containing protein 1 /MA7D1	Q3KQU3	MAP7D1	93106	63	19/75	16			0.491	0.150	ns
Eukaryotic translation initiation factor 3 subunit B /EIF3B	P55884	EIF3B	92823	56	10/83	17			1.000	0.313	ns
Advillin /AVIL	O75366	AVIL	92312	63	18/99	20			1.000	0.553	ns
Semaphorin-3D /SEM3D	O95025	SEMA3D	90735	57	15/88	14			1.000	0.313	ns
Zinc finger protein 585A /Z585	Q6P3V2	ZNF585A	90540	56	17/85	20			1.000	0.313	ns
Semaphorin-3E /SEM3E	O15041	SEMA3E	90368	69	13/62	14			1.000	0.313	ns
Oxysterol-binding protein 1 /OSBP1	P22059	OSBP	90220	85	22/87	19			1.000	0.313	ns
Integrin beta-3 /ITB3	P05106	ITGB3	90194	149	19/65	19			0.586	0.413	ns
Rabenosyn-5 /RBNS5	Q9H1K0	RBSN	89613	69	23/99	21			1.000	0.313	ns
Calcium-independent phospholipase A2-gamma /PLPL8	Q9NP80	PNPLA8	89219	70	22/97	22			1.000	1.000	ns
Zinc finger protein 700 /ZN700	Q9H0M5	ZNF700	89027	62	10/73	11			1.000	0.313	ns
KN motif and ankyrin repeat domain-containing protein 3 /KANK3	Q6NY19	KANK3	88999	67	18/99	17			1.000	1.000	ns
Coiled-coil alpha-helical rod protein 1 /CCHCR	Q8TD31	CCHCR1	88902	57	17/68	11			1.000	0.313	ns
Serine/threonine-protein kinase tousled-like 2 /TLK2	Q86UE8	TLK2	88405	71	18/62	17			1.000	0.313	ns
cTAGE family member 6 /CTGE6	Q86UF2	CTAGE6	88244	58	19/90	19			1.000	0.313	ns
Host cell factor 2 /HCFC2	Q9Y5Z7	HCFC2	87694	59	14/86	17			1.000	0.313	ns
FYVE, RhoGEF and PH domain-containing protein 4 /FGD4	Q96M96	FGD4	87598	66	15/69	16			1.000	0.313	ns
Zinc finger protein 366 /ZN366	Q8N895	ZNF366	86763	58	9/42	9			1.000	0.313	ns
Gelsolin /GELS	P06396	GSN	86043	78	11/57	10			1.000	0.553	ns
SNF-related serine/threonine-protein kinase /SNRK	Q9NRH2	SNRK	85136	59	13/62	18			1.000	0.313	ns
Krev interaction trapped protein 1 /KRIT1	O00522	KRIT1	84979	65	18/80	11			1.000	1.000	ns
MAP/microtubule affinity-regulating kinase 3 /MARK3	P27448	MARK3	84889	57	15/98	22			1.000	1.000	ns
Zinc finger protein 280C /Z280C	Q8ND82	ZNF280C	84867	57	10/48	15			1.000	0.313	ns

Myeloperoxidase /PERM	P05164	MPO	84784	69	18/99	19			0.745	0.513	ns
Hyaluronan mediated motility receptor /HMMR	O75330	HMMR	84448	67	22/96	26			1.000	0.553	ns
MAP/microtubule affinity-regulating kinase 4 /MARK4	Q96L34	MARK4	83267	63	19/90	23			1.000	0.313	ns
Ribosomal protein S6 kinase alpha-1 /KS6A1	Q15418	RPS6KA1	83070	58	10/52	14			1.000	0.313	ns
Centrosomal protein of 83 kDa /CEP83	Q9Y592	CEP83	83060	58	20/97	20			1.000	0.313	ns
Dynamin-1-like protein /DNM1L	O00429	DNM1L	82339	57	12/50	14			1.000	0.313	ns
Zinc finger protein 770 /ZN770	Q6IQ21	ZNF770	82008	58	13/79	14			1.000	0.313	ns
Eosinophil peroxidase /PERE	P11678	EPX	81958	60	8/40	9			1.000	0.313	ns
A-kinase anchor protein 17A /AK17A	Q02040	AKAP17A	81313	61	18/98	21			1.000	0.313	ns
Beta-adducin /ADDB	P35612	ADD2	81260	57	10/50	18			1.000	0.313	ns
Epidermal growth factor receptor kinase substrate 8-like protein 2 /ES8L2	Q9H6S3	EPS8L2	81197	57	11/52	11			1.000	0.313	ns
RUN and FYVE domain-containing protein 1 /RUFY1	Q96T51	RUFY1	80851	58	19/96	19			1.000	0.313	ns
ATP-binding cassette sub-family F member 3 /ABCF3	Q9NUQ8	ABCF3	80094	58	14/70	12			1.000	0.313	ns
Lactotransferrin /TRFL	P02788	LTF	80014	243	32/97	33			1.000	0.736	ns
Serotransferrin /TRFE	P02787	TF	79294	61	11/51	14			1.000	0.313	ns
Niban-like protein 2 /NIBL2	Q86XR2	FAM129C	78391	57	15/94	15			1.000	0.313	ns
NF-kappa-B-repressing factor /NKRF	O15226	NKRF	78308	58	13/69	16			1.000	0.313	ns
Erythrocyte membrane protein band 4.2 /EPB42	P16452	EPB42	77816	82	13/62	16			0.192	0.083	ns
Heterogeneous nuclear ribonucleoprotein M /HNRPM	P52272	HNRNPM	77749	96	25/98	31			0.491	0.150	ns
Tumor protein 63 /P63	Q9H3D4	TP63	77649	58	9/87	10			1.000	0.313	ns
F-BAR and double SH3 domains protein 1 /FCSD1	Q86WN1	FCHSD1	77180	60	14/70	14			1.000	0.313	ns
Vitamin K-dependent protein S /PROS	P07225	PROS1	77127	63	8/37	8			1.000	0.313	ns
Lebercilin-like protein /LCA5L	O95447	LCA5L	76857	66	19/62	18			1.000	0.313	ns
Leucine-rich repeat-containing protein 45 /LRC45	Q96CN5	LRRC45	76475	66	19/90	20			1.000	0.553	ns
Annexin A6 /ANXA6	P08133	ANXA6	76168	69	15/66	18			1.000	0.313	ns
Zinc finger matrin-type protein 1 /ZMAT1	Q5H9K5	ZMAT1	75734	60	12/55	16			1.000	0.313	ns
Peptide-N(4)-(N-acetyl-beta-glucosaminyl)asparagine amidase /NGLY1	Q96IV0	NGLY1	75484	67	14/70	14			1.000	0.313	ns
POZ-, AT hook-, and zinc finger-containing protein 1 /PATZ1	Q9HBE1	PATZ1	75438	59	7/34	13			1.000	0.313	ns
T-box transcription factor TBX2 /TBX2	Q13207	TBX2	75247	63	15/80	17			0.236	0.075	ns
E3 ubiquitin-protein ligase ZSWIM2 /ZSWM2	Q8NEG5	ZSWIM2	74624	62	17/84	17			1.000	0.313	ns
Kelch-like protein 13 /KLH13	Q9P2N7	KLHL13	74618	60	12/60	13			1.000	0.313	ns
Nucleolar GTP-binding protein 1 /NOG1	Q9BZE4	GTPBP4	74317	64	15/65	20			1.000	0.313	ns
Coiled-coil domain-containing protein 93 /CCD93	Q567U6	CCDC93	73437	56	15/86	22			1.000	0.313	ns
Katanin p80 WD40 repeat-containing subunit B1 /KTNB1	Q9BVA0	KATNB1	73257	58	8/33	8			1.000	0.313	ns
Netrin-4 /NET4	Q9HB63	NTN4	72761	60	9/70	8			1.000	0.313	ns
RNA polymerase II elongation factor ELL2 /ELL2	O00472	ELL2	72736	66	9/45	12			1.000	0.313	ns
Atypical kinase COQ8A, mitochondrial /COQ8A	Q8NI60	COQ8A	72418	59	10/47	14			1.000	0.313	ns
Endoplasmic reticulum chaperone BiP /BIP	P11021	HSPA5	72402	69	16/86	25			0.491	0.150	ns
Wee1-like protein kinase /WEE1	P30291	WEE1	72237	58	7/64	10			1.000	0.313	ns
5-aminolevulinate synthase, nonspecific, mitochondrial /HEM1	P13196	ALAS1	71391	61	9/55	9			1.000	0.313	ns
Albumin /ALBU	P02768	ALB	71317	244	30/96	46			0.586	0.413	ns
Cilia- and flagella-associated protein 100 /CP100	Q494V2	CFAP100	71301	78	20/82	23			1.000	0.639	ns
Actin-related protein 8 /ARP8	Q9H981	ACTR8	71294	63	9/33	9			0.491	0.150	ns
Acid-sensing ion channel 4 /ASIC4	Q96FT7	ASIC4	71144	58	12/77	14			1.000	0.313	ns
Heat shock cognate 71 kDa protein /HSP7C	P11142	HSPA8	71082	95	19/95	28			0.372	0.233	ns
E3 ubiquitin-protein ligase TRIM47 /TRI47	Q96LD4	TRIM47	70970	59	10/72	13			1.000	0.313	ns
Plastin-2 /PLSL	P13796	LCP1	70814	70	10/40	15			0.491	0.150	ns
Heat shock 70 kDa protein 1-like /HS71L	P34931	HSPA1L	70730	64	14/96	19			1.000	0.313	ns
RUN and FYVE domain-containing protein 2 /RUFY2	Q8WXA3	RUFY2	70707	68	14/83	21			1.000	1.000	ns
SH3 domain-containing protein 21 /SH321	A4FU49	SH3D21	70647	64	11/50	17			0.610	0.299	ns
BUD13 homolog /BUD13	Q9BRD0	BUD13	70535	57	15/62	18			1.000	0.313	ns
Formin-binding protein 1-like /FBP1L	Q5T0N5	FNBP1L	70478	64	20/96	24			1.000	1.000	ns
Probable ATP-dependent RNA helicase DDX41 /DDX41	Q9UJV9	DDX41	70477	62	17/93	22			1.000	0.313	ns
Collagen alpha-1(XIII) chain /CODA1	Q5TAT6	COL13A1	70363	58	7/21	12			1.000	0.313	ns
Heat shock 70 kDa protein 1A /HS71A	P0DMV8	HSPA1A	70294	60	13/96	20			1.000	0.313	ns
Heat shock 70 kDa protein 1B /HS71B	P0DMV9	HSPA1B	70294	60	13/96	20			1.000	0.313	ns
F-box/WD repeat-containing protein 1A /FBW1A	Q9Y297	BTRC	70248	61	10/41	14			1.000	0.313	ns
Endonuclease 8-like 3 /NEIL3	Q8TAT5	NEIL3	69265	57	8/61	15			1.000	0.313	ns
Zinc finger protein 791 /ZN791	Q3KP31	ZNF791	69108	70	14/76	23			0.236	0.075	ns
Medium-chain acyl-CoA ligase ACSF2, mitochondrial /ACSF2	Q96CM8	ACSF2	68993	61	8/44	12			1.000	0.313	ns
Zinc finger protein 248 /ZN248	Q8NDW4	ZNF248	68868	64	9/45	13			1.000	0.313	ns
Guanylate-binding protein 3 /GBP3	Q9H0R5	GBP3	68698	72	12/83	16			1.000	0.313	ns
Tyrosine-protein phosphatase non-receptor type 11 /PTN11	Q06124	PTPN11	68538	56	10/64	15			1.000	0.313	ns
Transketolase /TKT	P29401	TKT	68519	71	14/96	17			0.491	0.150	ns
Pescadillo homolog /PESC	O00541	PES1	68359	58	7/39	9			1.000	0.313	ns
Metal-response element-binding transcription factor 2 /MTF2	Q9Y483	MTF2	68301	63	15/61	20			1.000	1.000	ns
Phostensin /PPR18	Q6NYC8	PPP1R18	68187	59	15/80	18			1.000	0.313	ns
Cyclic nucleotide-binding domain-containing protein 2 /CNBD2	Q96M20	CNBD2	68038	61	17/98	19			1.000	0.313	ns
Moesin /MOES	P26038	MSN	67892	75	22/96	28			0.491	0.150	ns
Putative phosphatidylinositol 4-kinase alpha-like protein P2 /PI4P2	A4QPH2	PI4KAP2	67813	74	10/36	15			0.491	0.150	ns
Spastin /SPAST	Q9UBP0	SPAST	67497	58	8/61	13			1.000	0.313	ns
WD repeat-containing protein 1 /WDR1	O75083	WDR1	66836	65	14/98	18			1.000	0.639	ns

Transcriptional repressor p66-beta /P66B	Q8WXI9	GATAD2B	65562	58	16/59	16			1.000	0.313	ns
Coiled-coil domain-containing protein 38 /CCD38	Q502W7	CCDC38	65445	81	13/52	24			1.000	1.000	ns
WSC domain-containing protein 2 /WSCD2	Q2TBF2	WSCD2	64859	57	17/97	24			1.000	1.000	ns
Squalene monooxygenase /ERG1	Q14534	SQLE	64282	66	16/92	17			1.000	0.313	ns
FERM domain-containing protein 1 /FRMD1	Q8N878	FRMD1	63396	62	11/75	16			1.000	0.313	ns
Glucose-6-phosphate isomerase /G6PI	P06744	GPI	63335	114	19/98	25			1.000	1.000	ns
Stress-induced-phosphoprotein 1 /STIP1	P31948	STIP1	63227	62	17/78	27			1.000	0.313	ns
Phosphoglucomutase-like protein 5 /PGM5	Q15124	PGM5	62756	57	6/18	9			1.000	0.313	ns
SLAIN motif-containing protein 2 /SLAI2	Q9P270	SLAIN2	62733	68	14/96	18			1.000	0.313	ns
Doublesex- and mab-3-related transcription factor 2 /DMRT2	Q9Y5R5	DMRT2	62687	64	7/20	8			1.000	0.313	ns
Delta-1-pyrroline-5-carboxylate dehydrogenase, mitochondrial /AL4A1	P30038	ALDH4A1	62137	65	8/32	11			1.000	0.313	ns
Inactive phospholipase D5 /PLD5	Q8N7P1	PLD5	62015	57	7/64	13			1.000	0.313	ns
Breast carcinoma-amplified sequence 1 /BCAS1	O75363	BCAS1	61957	60	11/78	12			1.000	0.313	ns
Mitochondria-eating protein /MIEAP	Q8TC71	SPATA18	61926	56	13/67	17			1.000	0.313	ns
Testis-expressed basic protein 1 /TSBP1	Q5SRN2	TSBP1	61873	58	15/98	26			1.000	0.313	ns
Chondroitin sulfate N-acetylgalactosaminyltransferase 1 /CGAT1	Q8TDX6	CSGALNACT1	61769	76	11/51	14			1.000	0.313	ns
Src substrate cortact /SRC8	Q14247	CTTN	61720	71	16/96	21			1.000	0.313	ns
2-hydroxyacylsphingosine 1-beta-galactosyltransferase /CGT	Q16880	UGT8	61627	59	9/49	11			1.000	0.313	ns
Heparanase /HPSE	Q9Y251	HPSE	61395	58	11/72	19			1.000	0.313	ns
Zinc finger protein 775 /ZN775	Q96BV0	ZNF775	61310	58	12/98	14			1.000	0.313	ns
Ribosomal protein S6 kinase-like 1 /RPKL1	Q9Y6S9	RPS6KL1	60740	60	9/43	12			1.000	0.313	ns
Coronin-2A /COR2A	Q92828	CORO2A	60239	60	12/98	15			1.000	0.553	ns
Catalase /CATA	P04040	CAT	59947	197	25/98	42			1.000	0.761	ns
2'-5'-oligoadenylate synthase-like protein /OASL	Q15646	OASL	59702	76	10/79	12			1.000	0.313	ns
Ankyrin repeat and death domain-containing protein 1B /AKD1B	A6NHY2	ANKDD1B	59166	58	8/95	11			1.000	0.313	ns
Pyruvate kinase PKM /KPYM	P14618	PKM	58470	101	19/96	31			1.000	0.761	ns
WD40 repeat-containing protein SMU1 /SMU1	Q2TAY7	SMU1	58134	58	9/44	12			1.000	0.313	ns
Cell division cycle protein 20 homolog B /CD20B	Q86Y33	CDC20B	58040	66	6/34	10			1.000	0.313	ns
Nucleosome assembly protein 1-like 3 /NP1L3	Q99457	NAP1L3	57843	60	11/61	15			1.000	0.313	ns
Rab5 GDP/GTP exchange factor /RABX5	Q9UJ41	RABGEF1	57596	58	13/77	18			1.000	0.313	ns
Zinc finger and SCAN domain-containing protein 30 /ZSC30	Q86W11	ZSCAN30	57469	57	14/80	19			1.000	0.313	ns
TELO2-interacting protein 2 /TTI2	Q6NXR4	TTI2	57449	65	11/70	16			1.000	0.313	ns
Protein disulfide-isomerase A3 /PDIA3	P30101	PDIA3	57146	90	14/99	21			1.000	0.639	ns
Zinc finger protein 223 /ZN223	Q9UK11	ZNF223	57380	60	10/86	16			1.000	0.313	ns
Fibrinogen beta chain /FIBB	P02675	FGB	56577	268	28/99	54			0.327	0.190	ns
Nucleotidyltransferase MB21D2 /M21D2	Q8IYB1	MB21D2	56392	62	8/45	15			1.000	0.313	ns
Signal recognition particle 54 kDa protein /SRP54	P61011	SRP54	55953	57	16/97	28			1.000	0.313	ns
SH2 domain-containing adapter protein B /SHB	Q15464	SHB	55749	58	11/66	24			0.236	0.075	ns
Alpha-internexin /AINX	Q16352	INA	55528	62	6/16	12			1.000	0.313	ns
TNF receptor-associated factor 4 /TRAF4	Q9BUZ4	TRAF4	55218	58	10/51	16			0.491	0.150	ns
Probable asparagine--tRNA ligase, mitochondrial /SYNM	Q96I59	NARS2	54569	56	11/82	16			1.000	0.313	ns
Galactose-3-O-sulfotransferase 4 /G3ST4	Q96RP7	GAL3ST4	54417	61	9/98	12			1.000	1.000	ns
Solute carrier family 2, facilitated glucose transporter member /GTR3	P11169	SLC2A3	54345	63	8/41	15			0.491	0.150	ns
Uncharacterized protein C6orf118 /CF118	Q5T5N4	C6orf118	54308	59	16/97	24			1.000	0.313	ns
Interstitial collagenase /MMP1	P03956	MMP1	54144	68	14/85	18			1.000	1.000	ns
Dihydrolipoamide Branched Chain Transacylase E2 /ODB2	P11182	DBT	53852	66	9/41	13			1.000	0.313	ns
Vimentin /VIME	P08670	VIM	53676	75	14/79	31			1.000	1.000	ns
IQ domain-containing protein C /IQCC	Q4KMZ1	IQCC	53627	58	11/87	18			1.000	0.313	ns
Tyrosine--tRNA ligase, mitochondrial /SYYM	Q9Y2Z4	YARS2	53394	59	12/86	21			1.000	0.313	ns
Tripartite motif-containing protein 43B /TR43B	A6NCK2	TRIM43B	53246	64	11/61	18			1.000	0.313	ns
Protein RUFY3 /RUFY3	Q7L099	RUFY3	53216	57	14/78	20			1.000	0.313	ns
Cysteine protease ATG4C /ATG4C	Q96DT6	ATG4C	53034	63	8/56	17			1.000	0.313	ns
Clusterin /CLUS	P10909	CLU	53031	63	10/47	19			1.000	0.313	ns
Methanethiol oxidase /SBP1	Q13228	SELENBP1	52928	94	16/98	31			0.526	0.340	ns
N6-adenosine-methyltransferase non-catalytic subunit /MET14	Q9HCE5	METTL14	52688	63	7/32	10			0.491	0.150	ns
Fibrinogen gamma chain /FIBG	P02679	FGG	52106	181	19/84	33			1.000	1.000	ns
Kynurenine--oxoglutarate transaminase 3 /KAT3	Q6YP21	KYAT3	51824	60	13/97	22			0.491	0.150	ns
Mitochondrial import inner membrane translocase subunit TIM44 /TIM44	O43615	TIMM44	51666	64	9/55	14			1.000	0.313	ns
N-acetylgalactosamine kinase /GALK2	Q01415	GALK2	51258	64	13/82	19			1.000	0.313	ns
Tumor necrosis factor receptor superfamily member 10A /TR10A	O00220	TNFRSF10A	51254	60	5/42	11			1.000	0.313	ns
SH3 domain-binding protein 5 /3BP5	O60239	SH3BP5	50679	59	11/87	19			1.000	1.000	ns
B-cell linker protein /BLNK	Q8WV28	BLNK	50606	60	8/36	11			1.000	0.313	ns
PRAME family member 14 /PRA14	Q5SWL7	PRAMEF14	50484	58	10/73	17			1.000	0.313	ns
Adenylosuccinate synthetase isozyme 1 /PURA1	Q8N142	ADSS1	50462	80	15/97	27			1.000	0.313	ns
Fez family zinc finger protein 2 /FEZF2	Q8TBJ5	FEZF2	50034	63	10/99	23			1.000	0.313	ns
Meiosis-specific with OB domain-containing protein /MEIOB	Q8N635	MEIOB	50023	61	6/80	9			1.000	0.313	ns
Coiled-coil domain-containing protein 71 /CCD71	Q8IV32	CCDC71	49789	58	16/86	24			0.610	0.299	ns
Protein GOLM2 /GOLM2	Q6P4E1	GOLM2	49582	58	12/90	22			1.000	0.313	ns
R3H and coiled-coil domain-containing protein 1 /R3HC1	Q9Y3T6	R3HCC1	49460	64	11/73	15			0.491	0.150	ns
SPARC-related modular calcium-binding protein 1 /SMOC1	Q9H4F8	SMOC1	49443	61	7/49	12			1.000	0.313	ns
Radical S-adenosyl methionine domain-containing protein 1, mitochondrial /RSAD1	Q9HA92	RSAD1	49139	63	11/74	20			0.491	0.150	ns
Ras association domain-containing protein 8 /RASF8	Q8NHQ8	RASSF8	48526	76	15/99	31			1.000	0.639	ns

Ferrochelatase, mitochondrial /HEMH	P22830	FECH	48402	67	10/80	18			1.000	0.313	ns
Serine/threonine-protein kinase 25 /STK25	O00506	STK25	48310	65	6/22	17			1.000	0.313	ns
Transcription termination factor 3, mitochondrial /MTEF3	Q96E29	MTERF3	48055	57	7/49	15			1.000	0.313	ns
Actin-related protein 3 /ARP3	P61158	ACTR3	47797	82	14/99	35			1.000	1.000	ns
tRNA methyltransferase 10 homolog C /TM10C	Q7L0Y3	TRMT10C	47602	60	10/77	27			1.000	0.313	ns
43 kDa receptor-associated protein of the synapse /RAPSN	Q13702	RAPSN	47496	58	8/32	17			1.000	0.313	ns
Alpha-enolase /ENOA	P06733	ENO1	47481	111	18/98	34			0.773	0.564	ns
Flotillin-2 /FLOT2	Q14254	FLOT2	47434	56	8/43	19			1.000	0.313	ns
Beta-enolase /ENOB	P13929	ENO3	47299	60	10/80	20			0.491	0.150	ns
Terminal nucleotidyltransferase 5B /TET5B	Q96A09	TENT5B	47114	59	12/98	21			1.000	0.313	ns
Tubulin epsilon and delta complex protein 2 /TEDC2	Q7L2K0	TEDC2	47000	64	7/31	12			1.000	0.313	ns
Protein ZNF365 /ZN365	Q70YC5	ZNF365	46912	59	9/49	19			1.000	0.313	ns
Myeloid cell nuclear differentiation antigen /MNDA	P41218	MNDA	46092	64	8/69	20			0.704	0.444	ns
DnaJ homolog subfamily C member 28 /DJC28	Q9NX36	DNAJC28	45948	59	12/64	27			1.000	0.313	ns
28S ribosomal protein S29, mitochondrial /RT29	P51398	DAP3	45880	62	10/71	18			1.000	0.313	ns
Krueppel-like factor 12 /KLF12	Q9Y4X4	KLF12	44611	58	8/44	14			1.000	0.313	ns
p21-activated protein kinase-interacting protein 1 /PK1IP	Q9NWT1	PAK1IP1	44506	65	10/70	20			1.000	0.313	ns
Rab-interacting lysosomal protein /RILP	Q96NA2	RILP	44345	56	10/78	18			1.000	0.313	ns
Coiled-coil domain-containing protein 89 /CCD89	Q8N998	CCDC89	44181	56	9/44	22			0.491	0.150	ns
3-hydroxyisobutyryl-CoA hydrolase, mitochondrial /HIBCH	Q6NVY1	HIBCH	43797	57	10/64	26			0.491	0.150	ns
Probable G-protein coupled receptor 132 /GP132	Q9UNW8	GPR132	43497	64	6/37	11			1.000	0.313	ns
Suppressor APC domain-containing protein 2 /SAPC2	Q86UD0	SAPCD2	43066	60	10/70	24			1.000	0.313	ns
Pseudouridylate synthase RPUSD4, mitochondrial /RUSD4	Q96CM3	RPUSD4	42578	57	9/55	23			1.000	0.313	ns
Protein FAM81A /FA81A	Q8TBF8	FAM81A	42479	68	15/99	36			1.000	1.000	ns
Actin, aortic smooth muscle /ACTA	P62736	ACTA2	42381	132	15/60	32			0.704	0.444	ns
Actin, alpha skeletal muscle /ACTS	P68133	ACTA1	42366	131	15/60	32			0.704	0.444	ns
Actin, alpha cardiac muscle 1 /ACTC	P68032	ACTC1	42334	131	15/60	32			0.704	0.444	ns
Beta-actin-like protein 2 /ACTBL	Q562R1	ACTBL2	42318	88	11/98	22			0.173	0.102	ns
Actin, gamma-enteric smooth muscle /ACTH	P63267	ACTG2	42249	132	15/60	32			0.704	0.444	ns
Acidic fibroblast growth factor intracellular-binding protein /FIBP	O43427	FIBP	42137	58	7/96	14			1.000	0.313	ns
Zinc finger protein 781 /ZNT81	Q8N8C0	ZNF781	42127	60	10/49	24			1.000	0.313	ns
Actin, cytoplasmic 2 /ACTG	P63261	ACTG1	42108	244	22/99	45			1.000	0.313	ns
Actin, cytoplasmic 1 /ACTB	P60709	ACTB	42052	244	22/99	45			1.000	0.313	ns
Putative GRINL1B complex locus protein 2 /GCOM2	Q9BZD3	GCOM2	42029	54	8/45	17			1.000	0.313	ns
Endophilin-A2 /SH3G1	Q99961	SH3GL1	41692	58	10/83	16			1.000	0.313	ns
Synaptonemal complex central element protein 1 /SYCE1	Q8N0S2	SYCE1	40074	59	13/99	25			1.000	0.313	ns
26S proteasome non-ATPase regulatory subunit 8 /PSMD8	P48556	PSMD8	39872	73	10/64	20			1.000	0.313	ns
Fructose-bisphosphate aldolase A /ALDOA	P04075	ALDOA	39851	84	10/95	33			0.387	0.248	ns
Ubiquitin-associated domain-containing protein 2 /UBAC2	Q8NBM4	UBAC2	39337	58	6/35	15			1.000	0.313	ns
Coiled-coil domain-containing protein 68 /CCD68	Q9H2F9	CCDC68	39073	60	10/60	24			1.000	0.313	ns
Proline/serine-rich coiled-coil protein 1 /PSRC1	Q6PGN9	PSRC1	38887	68	7/34	16			0.491	0.150	ns
Golgi-resident adenosine 3',5'-bisphosphate 3'-phosphatase /IMPA3	Q9NX62	IMPAD1	38828	65	9/45	17			1.000	0.313	ns
Annexin A2 /ANXA2	P07355	ANXA2	38808	115	14/53	40			1.000	0.313	ns
Putative annexin A2-like protein /AXA2L	A6NMY6	ANXA2P2	38806	65	10/53	28			1.000	0.313	ns
Sorting nexin-15 /SNX15	Q9NRS6	SNX15	38325	61	10/79	23			1.000	0.313	ns
Speriolin-like protein /SPC1L	Q9H0A9	SPATC1L	37932	62	10/85	25			1.000	0.313	ns
Transaldolase /TALDO	P37837	TALDO1	37688	66	13/97	26			0.491	0.150	ns
Peroxisome biogenesis factor 10 /PEX10	O60683	PEX10	37616	62	10/62	15			1.000	0.313	ns
Galactosylgalactosylxylosylprotein 3-beta-glucuronosyltransferase 3 /B3GA3	O94766	B3GAT3	37270	58	8/49	18			1.000	0.313	ns
L-lactate dehydrogenase A chain /LDHA	P00338	LDHA	36950	67	12/97	29			1.000	0.685	ns
L-lactate dehydrogenase B chain /LDHB	P07195	LDHB	36900	76	12/97	27			0.491	0.150	ns
Delta-aminolevulinic acid dehydratase /HEM2	P13716	ALAD	36728	67	10/90	26			1.000	1.000	ns
Aldo-keto reductase family 1 member B15 /AK1BF	C9JRZ8	AKR1B15	36685	62	10/84	26			1.000	0.313	ns
Lymphokine-activated killer T-cell-originated protein kinase /TOPK	Q96KB5	PBK	36404	57	10/77	18			1.000	0.313	ns
Glyceraldehyde-3-phosphate dehydrogenase /G3P	P04406	GAPDH	36201	110	17/97	37			1.000	0.785	ns
Protein FAM76A /FA76A	Q8TAV0	FAM76A	35996	58	12/94	32			1.000	0.313	ns
Malate dehydrogenase, mitochondrial /MDHM	P40926	MDH2	35937	63	10/98	29			0.491	0.150	ns
Lambda-crystallin homolog /CRYL1	Q9Y2S2	CRYL1	35909	60	6/32	23			1.000	0.313	ns
Histone H1oo /H1FOO	Q8IZA3	H1FOO	35792	59	13/90	26			1.000	0.313	ns
Mediator of RNA polymerase II transcription subunit 27 /MED27	Q6P2C8	MED27	35637	62	7/40	14			1.000	0.313	ns
Oxidoreductase NAD-binding domain-containing protein 1 /OXND1	Q96HP4	OXNAD1	35345	87	13/97	33			0.704	0.444	ns
Probable tRNA(His) guanylyltransferase /THG1	Q9NWX6	THG1L	35151	63	10/75	20			0.491	0.150	ns
Carbonic anhydrase 5A, mitochondrial /CAH5A	P35218	CA5A	34956	58	5/68	17			1.000	0.313	ns
L-serine dehydratase/L-threonine deaminase /SDHL	P20132	SDS	34945	65	11/71	24			0.491	0.150	ns
THAP domain-containing protein 11 /THA11	Q96EK4	THAP11	34776	68	11/90	23			1.000	0.313	ns
NADH-cytochrome b5 reductase 3 /NB5R3	P00387	CYB5R3	34441	56	9/97	32			1.000	0.313	ns
Actin-related protein 2/3 complex subunit 2 /ARPC2	O15144	ARPC2	34426	66	12/99	35			0.610	0.299	ns
TP53-target gene 5 protein /T53G5	Q9Y2B4	TP53TG5	34340	58	12/99	33			1.000	0.313	ns
Ankyrin repeat domain-containing protein SOWAHD /SWAHD	A6NJG2	SOWAHD	34010	59	10/81	28			1.000	0.313	ns
Peptidyl-prolyl cis-trans isomerase E /PPIE	Q9UNP9	PPIE	33695	57	8/35	18			1.000	0.313	ns
Methyltransferase-like protein 6 /MET6L	Q8TCB7	METTL6	33629	73	11/79	26			0.491	0.150	ns
Sulfotransferase 4A1 /ST4A1	Q9BR01	SULT4A1	33462	64	7/61	20			1.000	0.313	ns

Arginine and glutamate-rich protein 1 /ARGL1	Q9NWB6	ARGLU1	33197	57	14/80	37			1.000	0.313	ns
ADP/ATP translocase 3 /ADT3	P12236	SLC25A6	33073	57	7/40	21			1.000	0.313	ns
Tropomyosin alpha-3 chain /TPM3	P06753	TPM3	32987	87	17/98	34			0.728	0.484	ns
Tropomyosin beta chain /TPM2	P07951	TPM2	32945	75	15/98	29			1.000	0.639	ns
**Tropomyosin alpha-1 chain /TPM1**	P09493	TPM1	32746	67	15/91	34			0.028	0.033	**p < 0.05**
Purine nucleoside phosphorylase /PNPH	P00491	PNP	32325	67	9/54	26			0.610	0.299	ns
Erythrocyte band 7 integral membrane protein /STOM	P27105	STOM	31882	80	8/98	30			1.000	1.000	ns
Zinc finger protein 138 /ZN138	P52744	ZNF138	31598	66	9/31	27			1.000	0.553	ns
Ras suppressor protein 1 /RSU1	Q15404	RSU1	31521	63	10/98	21			1.000	0.313	ns
Tubulin polyglutamylase complex subunit 1 /TPGS1	Q6ZTW0	TPGS1	31484	64	5/12	14			1.000	0.313	ns
Collectin-10 /COL10	Q9Y6Z7	COLEC10	31084	64	8/35	19			1.000	0.313	ns
Triosephosphate isomerase /TPIS	P60174	TPI1	31057	81	11/99	39			1.000	0.553	ns
Apolipoprotein A-I /APOA1	P02647	APOA1	30759	77	12/74	35			1.000	1.000	ns
**Bisphosphoglycerate mutase /PMGE**	P07738	BPGM	30158	73	10/93	38			0.019	0.009	**p < 0.05**
Phosphoethanolamine/phosphocholine phosphatase /PHOP1	Q8TCT1	PHOSPHO1	30150	58	8/73	33			1.000	0.313	ns
Dehydrogenase/reductase SDR family member 4 /DHRS4	Q9BTZ2	DHRS4	29803	57	9/80	25			0.491	0.150	ns
Carbonic anhydrase 2 /CAH2	P00918	CA2	29285	65	8/93	33			1.000	0.553	ns
Cathepsin G /CATG	P08311	CTSG	29161	75	12/95	41			1.000	0.313	ns
Cytokine-inducible SH2-containing protein /CISH	Q9NSE2	CISH	28987	68	9/96	16			1.000	0.313	ns
Carbonic anhydrase 1 /CAH1	P00915	CA1	28909	110	13/69	59			0.526	0.340	ns
Tropomyosin alpha-4 chain /TPM4	P67936	TPM4	28619	206	25/98	54			0.412	0.273	ns
Nicotinamide/nicotinic acid mononucleotide adenylyltransferase 3 /NMNA3	Q96T66	NMNAT3	28475	56	7/51	23			1.000	0.313	ns
UL16-binding protein 3 /ULBP3	Q9BZM4	ULBP3	28273	59	5/24	23			1.000	0.313	ns
14-3-3 protein zeta/delta /1433Z	P63104	YWHAZ	27899	95	15/98	40			0.526	0.340	ns
Chymase /CMA1	P23946	CMA1	27763	58	4/43	24			1.000	0.313	ns
Myelin protein P0 /MYP0	P25189	MPZ	27709	58	7/64	27			1.000	0.313	ns
Eukaryotic translation initiation factor 4H /IF4H	Q15056	EIF4H	27425	57	10/59	26			0.491	0.150	ns
Acidic leucine-rich nuclear phosphoprotein 32 family member C /AN32C	O43423	ANP32C	26917	59	7/52	22			1.000	0.313	ns
Testis-expressed protein 35 /TEX35	Q5T0J7	TEX35	26900	56	14/80	35			1.000	0.313	ns
Fibroblast growth factor 8 /FGF8	P55075	FGF8	26737	59	5/38	18			1.000	0.313	ns
V-type proton ATPase subunit E 2 /VATE2	Q96A05	ATP6V1E2	26115	68	8/33	26			1.000	0.313	ns
Transmembrane protein 247 /TM247	A6NEH6	TMEM247	25550	56	7/36	26			1.000	0.313	ns
Adenylate kinase 4, mitochondrial /KAD4	P27144	AK4	25366	72	7/60	41			1.000	0.313	ns
Peroxiredoxin-6 /PRDX6	P30041	PRDX6	25133	64	10/86	37			0.491	0.150	ns
Synaptonemal complex central element protein 2 /SYCE2	Q6PIF2	SYCE2	25016	64	7/42	27			1.000	0.313	ns
Putative tripartite motif-containing protein 61 /TRI61	Q5EBN2	TRIM61	24943	57	7/55	17			1.000	0.313	ns
GrpE protein homolog 1, mitochondrial /GRPE1	Q9HAV7	GRPEL1	24492	58	9/64	29			1.000	1.000	ns
Zinc finger protein 525 /ZN525	Q8N782	ZNF525	24138	57	10/94	45			0.491	0.150	ns
Uncharacterized protein MISP3 /MISP3	Q96FF7	MISP3	24128	72	8/39	26			0.610	0.299	ns
Fibronectin type III domain-containing protein 5 /FNDC5	Q8NAU1	FNDC5	23872	64	6/37	19			1.000	0.313	ns
Alpha-1-acid glycoprotein 1 /A1AG1	P02763	ORM1	23753	63	6/26	26			1.000	0.313	ns
Glutathione S-transferase P /GSTP1	P09211	GSTP1	23569	70	7/30	5			1.000	0.313	ns
Ras-related protein Rab-24 /RAB24	Q969Q5	RAB24	23395	64	9/88	41			0.491	0.150	ns
Probable peptidyl-tRNA hydrolase /PTH	Q86Y79	PTRH1	23207	56	8/82	31			1.000	0.313	ns
Ras-related protein Rab-13 /RAB13	P51153	RAB13	22988	58	9/80	42			1.000	0.313	ns
Neutrophil gelatinase-associated lipocalin /NGAL	P80188	LCN2	22745	67	9/96	38			1.000	1.000	ns
Transgelin /TAGL	Q01995	TAGLN	22653	58	8/87	26			1.000	0.313	ns
Transgelin-2 /TAGL2	P37802	TAGLN2	22548	68	11/98	45			0.491	0.150	ns
H/ACA ribonucleoprotein complex subunit 1 /GAR1	Q9NY12	GAR1	22505	57	10/80	34			1.000	0.313	ns
Peptidyl-prolyl cis-trans isomerase F, mitochondrial /PPIF	P30405	PPIF	22368	57	7/74	25			1.000	0.313	ns
Receptor expression-enhancing protein 1 /REEP1	Q9H902	REEP1	22354	58	8/99	30			1.000	0.313	ns
Platelet glycoprotein Ib beta chain /GP1BB	P13224	GP1BB	22274	67	5/62	23			1.000	0.553	ns
Flavin reductase (NADPH) /BLVRB	P30043	BLVRB	22219	98	12/70	71			1.000	1.000	ns
Peroxiredoxin-2 /PRDX2	P32119	PRDX2	22049	190	14/89	48			1.000	0.685	ns
Adenylate kinase isoenzyme 1 /KAD1	P00568	AK1	21735	65	10/88	45			0.236	0.075	ns
Ras-related protein Rap-1A /RAP1A	P62834	RAP1A	21316	64	9/96	25			1.000	0.639	ns
Ras-related protein Rap-1b-like protein /RP1BL	A6NIZ1	N/A	21140	59	8/87	36			0.491	0.150	ns
Ras-related protein Rap-1b /RAP1B	P61224	RAP1B	21040	80	10/98	33			1.000	0.736	ns
Ferritin light chain /FRIL	P02792	FTL	20064	67	8/56	36			0.491	0.150	ns
Actin-related protein 2/3 complex subunit 4 /ARPC4	P59998	ARPC4	19768	63	9/84	41			1.000	0.313	ns
NADH dehydrogenase [ubiquinone] 1 alpha subcomplex assembly factor 2 /NDUF2	Q8N183	NDUFAF2	19844	82	8/96	30			1.000	0.553	ns
Cathelicidin antimicrobial peptide /CAMP	P49913	CAMP	19517	75	9/88	42			1.000	0.639	ns
Peptidyl-prolyl cis-trans isomerase A /PPIA	P62937	PPIA	18229	96	12/98	57			0.352	0.214	ns
Ubiquitin-like protein 4A /UBL4A	P11441	UBL4A	17823	53	6/35	33			1.000	0.313	ns
Uncharacterized protein KIAA1143 /K1143	Q96AT1	KIAA1143	17455	77	7/85	40			1.000	0.313	ns
Myosin light polypeptide 6 /MYL6	P60660	MYL6	17090	57	6/55	46			1.000	0.313	ns
Lysozyme C /LYSC	P61626	LYZ	16982	78	7/97	35			1.000	0.639	ns
Actin-related protein 2/3 complex subunit 5-like protein /ARP5L	Q9BPX5	ARPC5L	16931	59	7/59	39			1.000	0.313	ns
Ubiquitin-conjugating enzyme E2 variant 1 /UB2V1	Q13404	UBE2V1	16598	63	8/63	35			1.000	1.000	ns
Protein FAM183BP /F183B	Q6ZVS7	FAM183BP	16183	57	5/32	34			1.000	0.313	ns
Hemoglobin subunit delta /HBD	P02042	HBD	16159	107	8/32	55			0.785	0.584	ns
Hemoglobin subunit beta /HBB	P68871	HBB	16102	149	12/69	84			0.192	0.083	ns

Putative nucleoside diphosphate kinase /NDK8	O60361	NME2P1	15690	63	8/63	43			1.000	0.313	ns
Histone H3.1t /H31T	Q16695	H3-4	15613	63	9/96	38			1.000	0.313	ns
Histone H3.1 /H31	P68431	H3C1	15509	65	9/96	38			1.000	0.313	ns
Histone HIST2H3PS2 /H3PS2	Q5TEC6	H3-2	15478	60	8/96	47			1.000	1.000	ns
Histone H3.2 /H32	Q71DI3	H3C15	15436	65	9/96	38			1.000	0.313	ns
Hemoglobin subunit alpha /HBA	P69905	HBA1	15305	107	9/55	66			0.158	0.091	ns
Protein FAM74A4/A6 /FAM74	Q5TZK3	FAM74A4	15049	58	9/98	60			1.000	0.313	ns
Programmed cell death protein 5 /PDCD5	O14737	PDCD5	14276	58	6/42	52			1.000	0.313	ns
Histone H2B type 1-M /H2B1M	Q99879	H2BC14	13981	58	9/97	52			1.000	0.313	ns
Histone H2B type 1-B /H2B1B	P33778	HIST1H2BB	13942	69	8/96	57			0.491	0.150	ns
Developmental pluripotency-associated 5 protein /DPPA5	A6NC42	DPPA5	13489	62	7/82	44			1.000	0.313	ns
Uncharacterized protein C7orf66 /CG066	A4D0T2	C7orf66	13396	58	5/58	20			1.000	0.313	ns
Protein S100-A9 /S10A9	P06702	S100A9	13291	90	11/98	77			1.000	1.000	ns
Vesicle-associated membrane protein 5 /VAMP5	O95183	VAMP5	12968	56	6/61	37			1.000	0.313	ns
60S ribosomal protein L36 /RL36	Q9Y3U8	RPL36	12303	59	7/43	43			1.000	0.313	ns
C-X-C motif chemokine 6 /CXCL6	P80162	CXCL6	12175	68	6/39	45			1.000	1.000	ns
Putative uncharacterized protein encoded by LINC00523 /CN070	Q86TU6	LINC00523	12081	62	6/67	40			1.000	0.313	ns
Parvalbumin alpha /PRVA	P20472	PVALB	12051	82	11/90	61			1.000	0.313	ns
60S ribosomal protein L37 /RL37	P61927	RPL37	11299	63	8/67	56			1.000	1.000	ns
10 kDa heat shock protein, mitochondrial /CH10	P61604	HSPE1	10925	65	8/98	52			0.351	0.159	ns
Dynein light chain roadblock-type 2 /DLRB2	Q8TF09	DYNLRB2	10905	59	5/27	63			1.000	0.313	ns
Putative uncharacterized protein encoded by LINC00310 /CU082	P59036	LINC00310	7521	64	6/52	40			1.000	0.313	ns
Guanine nucleotide-binding protein G(I)/G(S)/G(O) subunit gamma-5 /GBG5	P63218	GNG5	7428	56	4/33	39			1.000	0.313	ns
Small integral membrane protein 39 /SIM39	A0A1B0GW54	SMIM39	6009	70	5/36	33			1.000	0.313	ns

Table of protein identifications in the total proteome dataset. Bars represent the number of cardioembolic or atherothrombotic thrombi in which the protein was identified. Statistical differences based on stroke etiology are represented by color bars: blue bars indicate proteins significantly identified in atherothrombotic thrombi; red bar indicates a protein significantly identified in cardioembolic thrombi; and green bars indicate no significant differences between cardioembolic and atherothrombotic thrombi in the identified protein. Statistical analysis were performed using the chi-square and Fisher's exact tests, with significance set at $p < 0.05$. a, Accession numbers from the UniProt database (https://www.uniprot.org/, accessed on 30 April 2025). b, Protein scores > 56 were considered significant ($p < 0.05$) based on peptide mass fingerprinting using the Mascot database search algorithm (Matrix Science, London, UK, https://www.matrixscience.com/, accessed on 30 April 2025).
